# The role of the private sector in noncommunicable disease prevention and management in low- and middle-income countries: a series of systematic reviews and thematic syntheses

**DOI:** 10.1080/17482631.2022.2156099

**Published:** 2023-01-02

**Authors:** Keiko Marshall, Philippa Beaden, Hammad Durrani, Kun Tang, Roman Mogilevskii, Zulfiqar Bhutta

**Affiliations:** a Dalla Lana School of Public Health, University of Toronto, Toronto, Canada; b Department of Medicine, University of Toronto, Toronto, Canada; c Institute of Health Policy, Management, and Evaluation, University of Toronto, Toronto, Canada; d Vanke School of Public Health, Tsinghua University, Beijing, China; e Institute of Public Policy and Administration, University of Central Asia, Bishkek, Kyrgyzstan; f Centre for Global Child Health, Hospital for Sick Children, Toronto, Canada; g Centre for Excellence in Women and Child Health and Institute of Global Health and Development, The Aga Khan University, Karachi, Pakistan

**Keywords:** Private sector, noncommunicable diseases, chronic disease, low- and middle-income countries, public-private partnerships, governance and policy, healthcare provision, innovation, knowledge educator, investment and finance

## Abstract

**Purpose:**

Conduct six systematic reviews investigating for-profit private sector roles in NCD prevention and management in low- and middle-income countries (LMICs) through our *a priori* framework’s pillars.

**Methods:**

Six systematic reviews and thematic syntheses were performed between March-August 2021, Six databases, websites of relevant organizations, and references lists of included studies were comprehensively searched. Studies published in English from 2000 onwards involving the pillar of interest, for-profit private sector, NCD prevention/management, and LMIC context were included. Results were synthesized using an inductive thematic synthesis approach.

**Results:**

Ultimately, 25 articles were included in the PPP review, 33 in Governance and Policy, 22 in Healthcare Provision, 15 in Innovation, 14 in Knowledge Educator, and 42 in Investment and Finance. The following themes emerged: PPPs (coordination; financial resources; provision; health promotion; capacity building; innovation; policy); Governance/Policy (lobbying; industry perception; regulation); Healthcare Provision (diagnosis/treatment; infrastructure; availability/accessibility/affordability); Innovation (product innovation; process innovation; marketing innovation; research; innovation dissemination); Knowledge Educator (training; health promotion; industry framework/guideline formation); Investment and Finance (treatment cost; regulation; private insurance; subsidization; direct investment; collaborative financing; innovative financing; research).

**Conclusion:**

These findings will be instrumental for LMICs considering private sector engagement. Potential conflicts of interest must be considered when implementing private sector involvement.

## Introduction

Noncommunicable diseases (NCDs) are a pressing global health issue. In 2019, they were the top cause of death worldwide, resulting in 42.0 million deaths (Institute for Health Metrics and Evaluation [IHME], 2020). The four main types of NCDs are cardiovascular disease (CVD), cancers (neoplasms), chronic respiratory diseases, and diabetes (WHO, 2021). Low- and middle-income countries (LMICs) are disproportionately impacted by NCDs, accruing over three-quarters of global NCD deaths, and 85% of premature deaths from NCDs (WHO, 2021). The high rates of death due to NCDs threaten progress towards achieving the sustainable development goals (SDGs), specifically 3.4 targeting a 1/3 reduction in premature deaths from NCDs (WHO, 2021).

NCD risk factors are multisectoral in origin, including unhealthy diets, physical inactivity, exposure to tobacco smoke, and harmful use of alcohol. Thus, an “all-of-society” approach including the private sector is necessary (Prescott & Stibbe, 2017; WHO, 2021). The World Health Organization (WHO) Global Action Plan for the Prevention and Control of NCDs 2013–2020 suggests that multi-stakeholder engagement including the private sector is required for effective NCD prevention and management (WHO, 2013). Furthermore, the U.N. Political Declaration of the High-Level Meeting of the General Assembly on the Prevention and Control of NCDs recommended engaging non-health actors and key stakeholders, including the private sector, in collaborative partnerships to promote health and reduce NCD risk factors (U.N. General Assembly, 2012).

The private sector plays a significant role in health systems as a solution to challenges including changes in disease burdens, budgetary constraints, and demographic shifts (Clarke & Paviza, 2018). Over time, the private for-profit sector specifically has situated itself as a driver of innovation and provider of high-quality healthcare, contributing to the provision of health-related services and products, funding and investment, workforce training, and infrastructure support (Clarke & Paviza, 2018; Clarke et al., 2019).

There is a lack of evidence and guidance, however, regarding the role that the for-profit private sector can play in NCD prevention and management (Clarke et al., 2019). To fill this gap, we previously developed an *a priori* framework outlining how the private for-profit sector is involved in NCD prevention and management. The framework contains 6 pillars: public-private partnerships (PPPs); governance and policy; healthcare provision; innovation; knowledge educator; investment and finance. This study aimed to conduct a systematic review for each pillar to better understand how the private sector acts within these roles, specifically in an LMIC context.

## Methods

Six different systematic reviews were conducted, all of which are reported in accordance with the Preferred Reporting Items for Systematic Reviews and Meta-analysis (PRISMA). No protocol was prepared.

### Search strategy

Comprehensive searches of Embase, PubMed, Web of Science, Cochrane Library, ProQuest ABI/Inform, and Business Source Premier were conducted in March 2021 for each of the six reviews. Websites of the following relevant organizations were also searched in April-July 2021 to identify grey literature: WHO, World Bank, United Nations Children’s Fund (UNICEF), Organization for Economic Co-operation and Development (OECD), American Cancer Society, NCD Alliance, Union for International Cancer Control (UICC), Center for Strategic and International Studies (CSIS), World Economic Forum, Centers for Disease Control and Prevention (CDC), Harvard School of Public Health, United States Agency for International Development (USAID), Medtronic, Astra Zeneca, Novo Nordisk, Rabin Martin, C3, and Merck. Results were filtered to only include articles published from the year 2000 onwards, in English.

A search strategy including the phrases “private sector”, “framework pillar of interest” and “NCDs” was formulated and administered, which is available in Appendix A.

### Study selection

Studies were considered for inclusion if their focus was the framework pillar of interest, the for-profit private sector, and NCD prevention and management in an LMIC context. News articles, press releases, presentations, or articles unavailable as full text were excluded. Covidence Software (2021) was used for screening by one reviewer in each review. Reference lists of included studies were additionally screened for relevant studies.

### Data extraction

One reviewer conducted data extraction using Covidence Software (2021) and a pre-piloted data extraction form for each review. The complete data extraction form is available in Appendix B.

### Quality assessment

The quality of each study was evaluated by the same reviewer using the tool developed by Hawker et al (Hawker et al., 2002). which assesses nine areas for quality: abstract and titles; introduction and aims; method and data; sampling; data analysis; ethics and bias; results; transferability and generalizability; implication and usefulness. Each section was awarded a score of 4=good, 3=fair, 2=poor, or 1=very poor. Values were added up to provide a total score, which corresponds to a letter grade: 30-36=A=high quality; 24-29=B=medium quality; 9-24=C=low quality.

### Data synthesis

An inductive thematic synthesis method was used (Thomas & Harden, 2008). Extracted data was systematically coded in accordance with its meaning to translate findings into a universal code. Codes were then categorized to elucidate overarching themes that existed in the included studies for each review. These themes were used to answer the proposed research question regarding the private sector’s role in NCD prevention and management in LMICs.

### Patient and public involvement

Neither patients nor public were involved in the design, conduct, reporting, or dissemination plans of our research.

## Results

**Figure 1.**
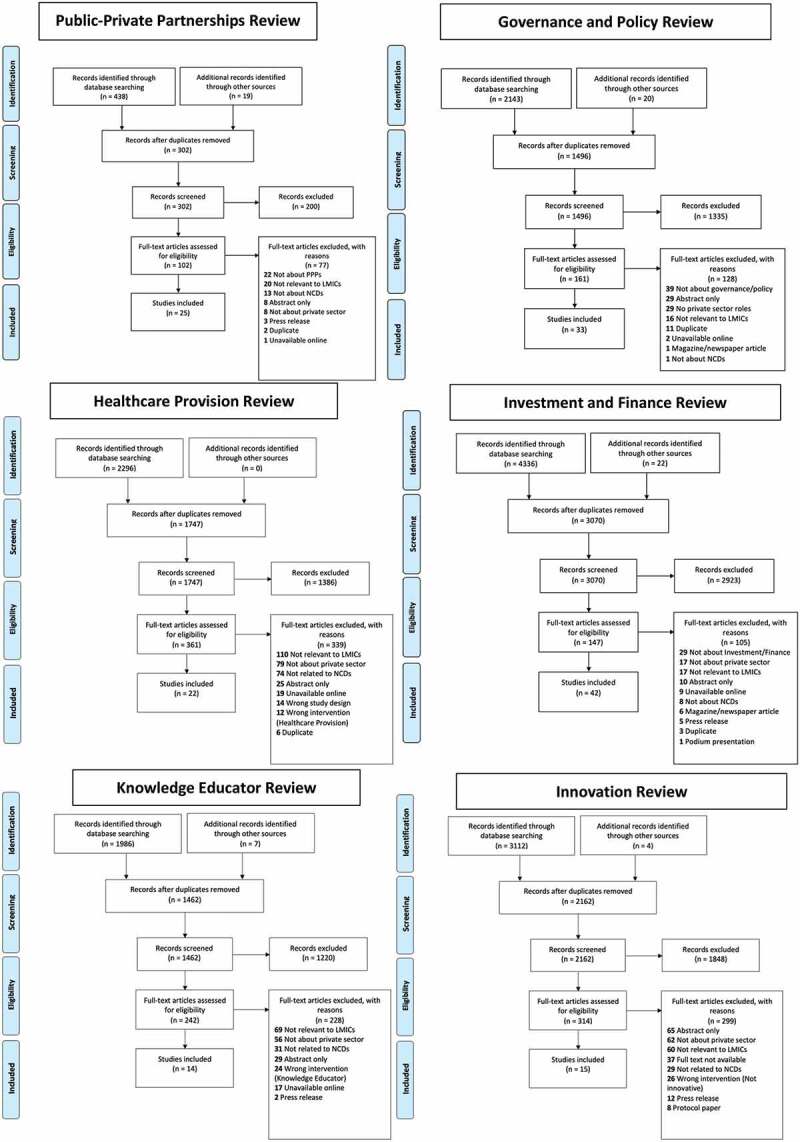
PRISMA flow diagrams for each of the 6 systematic reviews.

Following the removal of duplicates, 302 articles were screened for inclusion in the PPP review, 1496 for Governance and Policy, 1747 for Healthcare Provision, 2162 for Innovation, 1462 for Knowledge Educator, and 3070 for Investment and Finance (Figure 1). Of these, 102 underwent full-text screening in the PPP review, 161 in Governance and Policy, 361 in Healthcare Provision, 314 in Innovation, 242 in Knowledge Educator, and 147 in Investment and Finance. Ultimately, 25 articles were included in the PPP review, 33 in Governance and Policy, 22 in Healthcare Provision, 15 in Innovation, 14 in Knowledge Educator, and 42 in Investment and Finance. Appendix C contains characteristics of the included studies.

Several themes were elucidated in each review that describe the for-profit private sector’s role in NCD prevention and management in LMICs.

### Public-private partnerships

In the PPP review, seven themes were elucidated: coordination; provision; financial resources; health promotion; innovation; capacity building; policy (Table 1).

**Table I.** Themes and their associated sub-themes and components elucidated in each of the six systematic reviews conducted. These themes and sub-themes describe the private sector’s role in NCD prevention and management through each of the review topics.

Themes	Sub-Themes	Components
**Public-Private Partnerships**		
Coordination	Align goals	• Platform for dialogue • Collaboration
	Leverage expertise	• Private sector-specific skills • Private sector expertise in areas public sector lacks
Financial Resources	Mobilize funds	• Financial donations • Corporate social responsibility spending • Private financing and investment
Provision	Medicine provision	• Increase access to medicine • Improve prices of medicine available
	Service provision	• Improve quality of service • Increase service coverage and access
Health Promotion	Health education	• Behaviour change • Increase awareness and reduce stigmas
	Physical activity	• Promote physical activity
	Nutrition	• Improve access to better nutrition
Capacity Building	Training	• Provide training and educational materials to healthcare providers
Innovation	Research	• Leverage public and private research capacity and funding • Encourage reformulation
	Technology	• Improve access use of health technology
Policy	Policy influencer	• Raise profile of NCDs • Provide recommendations
**Governance and Policy**		
Lobbying	Fight policy	• Weaken policies and regulation • Prevent effective policy implementation
	Legal strategies	Litigation
	Policy influencer	• Prepare policy drafts • Influence policy discourse
	Government infiltration	• Hijack government representation • Build relationships, revolving door • Fund campaigns
	Collaboration	• PPPs • Joint efforts
Industry perception	Framing	• Manipulate public opinion • Media capture • Hire experts
	Shape evidence	• Fund research studies • Build research centres • Discredit science
	Economic importance	• Employment • Economic growth • Trade
Regulation	Self-regulation	• Voluntary self-regulationPolicy substitutions
	Evade regulation	• Bribes and sponsorshipsPolicy loopholes
		**Healthcare Provision**
Diagnosis and treatment		• Provide primary healthcare servicesProvide specialized healthcare servicesGreater diagnosis and management of NCDs
Infrastructure		• Provide infrastructure like hospitals, laboratories, pharmaciesPrivate facilities better equipped to manage NCDs
Availability, accessibility, and affordability		• Increase availability and accessibility of healthcare servicesInitiatives to improve affordability (insurance, mitigate cost of services)
**Innovation**		
Product innovation	Medications	• Introduction of new or significantly improved medication
	Health Information System (HIT) and Information Communication Technology (ICT)	• Information technology systems to collect and transfer dataImprove monitoring of interventions, NCD burdens
	Technology	• Anticipate trends and develop innovative technology as needed
	Upgrade of existing technologies	• Improve existing tools, devices, and technology to enhance services
Process innovation	Increasing outreach	• More efficient delivery methods for NCD interventions
	Private healthcare insurance	• Innovative health insurance programs including health promotion
Marketing innovation	Tailoring services	•Innovative pricing of products tailored to socioeconomic status of each LMIC
Research		• Create new and/or significantly improved products and services • Monetary support • In-kind support (participants, laboratories, data, expertise)
Innovation dissemination		• Promoting innovation to target populations for adoption • Fund publications to reach greater audiences • Educational grants to promote research
**Knowledge Educator**		
Training	Building research capacity	• Invest in research capacity • Train and provide mentorship to local researchers
	Building health workforce capacity	• Knowledge and skills advancement • Training in medical devices and processes
Health promotion	Awareness	• Remove barriers to access information • Educational programs to improve awareness of NCDs and their prevention/treatment
	Accessibility	• Increase access to healthy activities • Increase access to health knowledge
Industry framework and guideline formation		• Educate healthcare providers on international guidelines and standards • Develop standard protocols based on emerging research
**Investment and Finance**		
Cost of treatment	High prices	• Price of medicine and service higher in private than public sector
	OOP expenditure	• OOP spending higher in private than public sector
	Supply chain & manufacturer pricing	• Transport, storage, processing, packaging add-on costs • Inflated prices, largest contributor to final price
	Mark-ups	• Retail mark-ups high in private sector
Regulation	Self-regulation	• Promote standard prices
	Taxes	• Generate revenue to fund NCD care
Health insurance	Private insurance	• Barrier to care—high administrative costs • Reduce OOP expenses • Relieve financial pressure on government health budgets
Subsidization	Subsidies	• Improve affordability of services that contribute to NCD mitigation (i.e., gym memberships, healthy foods, medicines)
Direct investment	Private investment	• Fund NCD service delivery and expensive treatments, fill financing gaps • Corporate social responsibility spending • “Best buys”
Collaborative financing	Multi-sector collaboration	• Capitalize on individual strengths and ensure evenly distributed funds • Aligned objectives
	PPPs	• Leverage public and private investment
Innovative financing	Innovative programs	• Micro-levies • Credit card rounding plans • Mobile phone applications
	Development bonds	• Secure capital from private investors • Incentivize rapid results
	Development bank lending	• Promote private investment
	MDTF	• Engage private sector to respond to fiscal demand
Research	Originator vs. generic	• Develop new formulations of patent-restricted originator brand products • Originator products more expensive than generic equivalents • Intellectual property
	Research funding	• Invest in innovation • Potential biases in results

#### Coordination

Coordination includes how PPPs coordinate the private and public sectors to produce the most beneficial result. This occurs through aligning private and public sector goals (Hospedales & Jané-Llopis, 2011; Kraak et al., 2012; UNICEF, 2020; WHO GCM/NCD Working Group, 2018) and leveraging the skills and expertise from both sectors, specifically complimenting areas the public sector lacks (Hawkes & Buse, 2011; Johnson et al., 2018; Jones, 2021; Oluwole & Kraemer, 2013; Ota et al., 2018; Prescott & Stibbe, 2017; Silva et al., 2017; Thow, Verma, etal., 2018; UNICEF, 2019, 2020; WHO, 2016, 2019; World Economic Forum & PAHO, 2013). Transactional partnerships build mutually beneficial relationships through compatible goals, advancing each partner’s agenda (Kraak et al., 2012).

#### Financial resources

The private sector provides financial resources through PPPs directed towards NCD mitigation efforts. PPPs mobilize resources that finance NCD interventions, programming, infrastructure, and research (Alizadeh et al., 2020; Healthy Caribbean Coalition & NCD Alliance, 2017; Johnson et al., 2018; Jones, 2021; Kraak et al., 2012; Moscetti & Taylor, 2015; Oluwole & Kraemer, 2013; Ota et al., 2018; Trager, 2020; UNICEF, 2019, 2020; WHO GCM/NCD Working Group, 2018; World Economic Forum & PAHO, 2013). Philanthropic partnerships facilitate financial donations from private partners (Kraak et al., 2012). Transformational partnerships are more complex, with greater resource investment such that partner cultures and practices are influenced (Kraak et al., 2012).

#### Provision

The theme of provision pertains to private partners within PPPs aiding in the provision of affordable and high-quality treatment and services for NCDs. This is through the donation of treatment and service, or contractual agreements. PPPs increase access to safe, effective, and affordable NCD medication and health technology (Das et al., 2017; Goroff & Reich, 2010; Johnson et al., 2018; Oluwole & Kraemer, 2013; Shannon et al., 2019; WHO GCM/NCD Working Group, 2018). They do this by reforming the market, storage, and supply chain by involving private partners like the pharmaceutical industry (Thow, Verma, etal., 2018). Strategies encouraging private sector participation, like PPPs, can also yield higher quality NCD-related health services by increasing coverage and quality of NCD screening, diagnosis, and treatment (Alizadeh et al., 2020; Goroff & Reich, 2010; Hospedales & Jané-Llopis, 2011; Johnson et al., 2018; Jones, 2021; Oluwole & Kraemer, 2013; Shannon et al., 2019; Trager, 2020).

#### Health promotion

Health promotion includes public education about NCDs and their risk factors and promoting healthier habits. Through PPPs the private sector facilitates population education interventions regarding NCD screening, prevention, treatment, and diagnosis (Kraak et al., 2012; Oluwole & Kraemer, 2013; Shannon et al., 2019) encourages physical activity (Healthy Caribbean Coalition & NCD Alliance, 2017; Silva et al., 2017; World Economic Forum & PAHO, 2013) and improves access to nutritious foods (Thow, Verma, etal., 2018; WHO, 2013). This upstream approach helps improve NCD burdens in LMICs through prevention.

#### Capacity building

Capacity building includes training healthcare professionals in NCD mitigation techniques. Through PPPs the private sector facilitates training sessions that disseminate private sector skills, improving quality and capacity of local health systems for NCD mitigation (Johnson et al., 2018; Jones, 2021; Oluwole & Kraemer, 2013; Ota et al., 2018; Prescott & Stibbe, 2017; Shannon et al., 2019; WHO, 2013). PPPs provide capacity support and implementation research which improve NCD service delivery (Johnson et al., 2018).

#### Innovation

Innovation includes research and the distribution of technology relating to NCDs. Through PPPs, private partners facilitate data sharing, contribute to NCD-related research initiatives (Moodie et al., 2013; Moscetti & Taylor, 2015; Silva et al., 2017; Trager, 2020; WHO GCM/NCD Working Group, 2018) and facilitate access to innovative NCD-related technology (Johnson et al., 2018; Kraak et al., 2012; Prescott & Stibbe, 2017; WHO GCM/NCD Working Group, 2018).

#### Policy

The private sector plays a role in shaping policies relating to NCD prevention and management. Through PPPs, the private sector is given an enhanced opportunity to contribute to policy discussions that will govern NCD mitigation strategies globally and in LMICs, although this involvement is heavily prone to conflicts of interest (Moscetti & Taylor, 2015).

### Governance and policy

In the Governance and Policy review, three themes were elucidated: lobbying; industry perception; regulation (Table 1).

#### Lobbying

The private sector lobbies governments and other governing bodies. These efforts include industries fighting policy implementation (Carriedo et al., 2021; Casswell, 2019; Coriakula et al., 2018; Gortmaker et al., 2012; Gómez, 2019; Jaichuen et al., 2018; Khayatzadeh-Mahani et al., 2018; Mariath & Martins, 2020; Mialon et al., 2016, 2020, 2021; Oladepo et al., 2018; Sanni et al., 2018; Tangcharoensathien et al., 2019; Thow, Greenberg, etal., 2018; WHO, 2020, 2021; Williams, 2015) applying legal strategies such as litigation (George, 2018; Jaichuen et al., 2018; Mialon et al., 2016, 2021; Tangcharoensathien et al., 2019) and acting as policy influencers in the policymaking process (Buse et al., 2017; Carriedo et al., 2021; Casswell, 2013, 2019; George, 2018; Khayatzadeh-Mahani et al., 2018; Mialon et al., 2020; Moodie et al., 2013; Moscetti & Taylor, 2015; Myers et al., 2017; Suzuki et al., 2021; Thow, Greenberg, etal., 2018; WHO, 2021; Williams, 2015). Lobbying also includes the infiltration of government representation through relationship building and financial incentives (Carriedo et al., 2021; Casswell, 2019; George, 2018; Gómez, 2019; Mialon & Da Silva Gomes, 2019; Mialon et al., 2016, 2020; Tangcharoensathien et al., 2019) and collaboration through methods like PPPs (Bergman et al., 2012; Buse et al., 2017; Casswell, 2019; Mialon et al., 2016, 2021; Moodie et al., 2013; Moscetti & Taylor, 2015; WHO, 2016). Lobbying activities can shape the NCD-related governance and policy environment.

#### Industry perception

Public and government perception of industries impacts NCD-related governance and policies involving the private sector, and their level of involvement in policymaking. The private sector thus attempts to control this perception through framing (including corporate social responsibility activities, media capture, hiring prominent experts) (Casswell, 2013, 2019; George, 2018; Gómez, 2019; Jaichuen et al., 2018; Mialon & Da Silva Gomes, 2019; Mialon et al., 2016, 2020; Moodie et al., 2013; Moscetti & Taylor, 2015; Myers et al., 2017; Oladepo et al., 2018; Suzuki et al., 2021; Thow, Greenberg, etal., 2018) shaping scientific evidence that informs policies (George, 2018; Gómez, 2019; Jaichuen et al., 2018; Mialon & Da Silva Gomes, 2019; Mialon et al., 2016, 2020; Moodie et al., 2013; Moscetti & Taylor, 2015; Myers et al., 2017; Tangcharoensathien et al., 2019) and emphasizing industry’s economic importance (Carriedo et al., 2021; George, 2018; Jaichuen et al., 2018; Mialon & Da Silva Gomes, 2019; Mialon et al., 2016, 2020, 2021; Thow, Verma, etal., 2018, Thow, Greenberg, etal., 2018; WHO, 2021; Williams, 2015). This paints the private sector in a more positive light and as important stakeholders in economies and NCD policy to preserve their involvement in policy discussions and limit regulations.

#### Regulation

Regulation involves the governance strategies in place that aim to improve NCD burdens within countries by regulating potentially harmful activities and products. The private sector plays a role in their own regulation via voluntary self-regulation and company-mandated policies (Buse et al., 2017; Casswell, 2013; Cetthakrikul et al., 2019; George, 2018; Jaichuen et al., 2018; Lauber et al., 2020; Mialon & Da Silva Gomes, 2019; Mialon et al., 2016, 2020, 2021; Moodie et al., 2013; Moscetti & Taylor, 2015; Myers et al., 2017; Sacks et al., 2015; Suzuki et al., 2021; WHO, 2020). The private sector also, however, employs strategies to avoid regulation implemented by other governing bodies (Ben Romdhane et al., 2015; Bhojani et al., 2014; Coriakula et al., 2018; WHO, 2021).

### Healthcare provision

In the Healthcare Provision review, three themes were elucidated: availability, accessibility, and affordability; diagnosis and treatment; infrastructure (Table 1).

#### Diagnosis and treatment

Diagnosis and treatment (therapy) refers to the role of the private sector in diagnosing and treating NCDs in LMICs. The private sector is the main provider of primary healthcare services for NCD treatment in many LMICs (Berendes et al., 2011; Bigdeli et al., 2016; Elias et al., 2018; Mukherjee et al., 2011). They are often depended on for more specialized NCD care (Elias et al., 2018; Lewis et al., 2015; Pandian et al., 2007) are more likely to diagnose and manage NCDs compared to public health systems (Cataife, 2012) and have a greater ability to provide access to necessary medications (Bhuvan et al., 2019; Bissell et al., 2016; Syed et al., 2018).

#### Infrastructure

The private sector in LMICs contributes to NCD management by offering infrastructure such as hospitals, laboratories for testing, and pharmacies beyond those offered by the government (Angwenyi et al., 2020; Ben Romdhane et al., 2015; Polanczyk & Ribeiro, 2009; Rawal et al., 2020; Wearne et al., 2019). Private sector infrastructure is also often more equipped and ready to provide general and more specialized NCD-related care (Angwenyi et al., 2020; Bhojani et al., 2012; Bintabara et al., 2018; Cataife, 2012; Polanczyk & Ribeiro, 2009; Smith & Rabadan-Diehl, 2016; Wearne et al., 2019).

#### Availability, accessibility, and affordability

The role of the private sector in healthcare provision increases the availability and accessibility of NCD services in LMICs (Agarwal, 2005; Ashigbie et al., 2020; Atre, 2015; Bhojani et al., 2012; Mukherjee et al., 2011; Musinguzi et al., 2015; Wearne et al., 2019). This increased availability is often less affordable (Balasubramaniam et al., 2014; Subramanian et al., 2018; Wirtz et al., 2018) however, the private sector is involved in other initiatives that aim to improve affordability such as private medical insurance or mitigating the cost of treatment or transport (Abraham et al., 2009). The availability of generic and originator medicines in private pharmacies provide patients the opportunity to buy medications within their budget (Dabare et al., 2014).

### Innovation

In the Innovation review, five themes were elucidated: product innovation; process innovation; marketing innovation; research; innovation dissemination (Table 1).

#### Product innovation

Product innovation involves the introduction of new or significantly improved goods or services for NCD prevention and management (i.e., polypills, improved distribution methods) (Hancock et al., 2011; Nari Kahle et al., 2013; Plum et al., 2020; U.S. Department of State, 2011) and development of new health information or communication technology systems to improve data collection and monitoring (Doshi et al., 2021; Engelgau et al., 2016; Ganju et al., 2020; Global Systems for Mobile Communications, 2019; Rouleau et al., 2017; Shuvo et al., 2015; WHO, 2008). It also involves anticipating NCD-related needs and developing innovative technology that meets them (da Cruz Paula et al., 2020) and the upgrading of existing tools, devices, and technology to enhance services (Checkley et al., 2014; IFC, 2011; U.S. Department of State, 2011).

#### Process innovation

Process innovation pertains to the implementation of new or improved production or delivery methods by the private sector. This involves novel models of care that increases outreach for NCDs through novel service delivery packages (Allotey et al., 2014; Herzlinger, 2006; van de Vijver et al., 2013) and innovative private health insurance programs that include health promotion interventions (El-Sayed et al., 2015; Lambert & Kolbe-Alexander, 2013).

#### Marketing innovation

Marketing innovation is the implementation of new marketing methods with significant changes in aspects such as product design, packaging, placement, promotion, or pricing. In LMICs, the private sector has innovated ways to tailor NCD services and their prices depending on the socioeconomic status of countries so as to improve accessibility and affordability (AstraZeneca, 2021; Nari Kahle et al., 2013; Novo Nordisk, 2017; Rockers et al., 2019; Shannon et al., 2019; U.S. Department of State, 2011).

#### Research

Research is an important process in innovation. Basic and applied research are two processes through which new and/or significantly improved services/products are created and channelled into technological innovations (Omachonu & Einspruch, 2010). The private sector is heavily involved in research that is novel in nature, providing monetary or in-kind (participants, laboratories, data, expertise) support (Grancelli, 2005; Saldarriaga et al., 2017; Medtronic Foundation n.d.). Research capacity in LMICs is poor (Engelgau et al., 2016) thus the private sector supporting research are additionally contributing to capacity building (Engelgau et al., 2016; Medtronic Foundation n.d.).

#### Innovation dissemination

Innovation dissemination explains how a new product or idea gains momentum over time and diffuses within a population or social system (Dearing & Cox, 2018). When promoting innovation, one must understand the target population and any factors that facilitate or hinder its adoption. The private sector may fund publications to ensure research reaches a greater audience (Tzeel, 2011) or provide educational grants to promote involvement in research studies (Evert et al., 2019).

### Knowledge educator

In the Knowledge Educator review, three themes were elucidated: training; health promotion; industry framework and guideline formation (Table 1).

#### Training

The private sector contributes knowledge regarding NCD prevention and management through the provision of training. Research is an integral component of combating NCDs in LMICs, but research capacity is suboptimal (Bloomfield et al., 2016; Ebrahim et al., 2013; Malekzadeh et al., 2020). Private sector investment in research capacity (Bloomfield et al., 2016; Engelgau et al., 2016; Malekzadeh et al., 2020) and provision of training and mentorship to local researchers (Engelgau et al., 2016) are fundamental in addressing NCDs. LMICs also often have limited healthcare providers available to combat NCDs (Joshi et al., 2014; Pastakia et al., 2020; Pati et al., 2020) thus the private sector has provided training to healthcare providers for knowledge and skill advancement, and the use of new medical devices and processes (Merck , 2021; Pastakia et al., 2020; Ratzan et al., 2013; AVON Global Scholars, n.d.; Reeves, 2011).

#### Health promotion

Health promotion is the process of enabling people to increase control over and improve their own health by equipping them with the skills and capabilities to do so (Dobe, 2012; Pati et al., 2020). The private sector has promoted health through educational programs that remove barriers to accessing health information and improve awareness (Laar et al., 2019; Novo Nordisk, 2017; Ratzan et al., 2013) and programs that increase access to healthy activities and health knowledge (Patel et al., 2010).

#### Industry framework and guideline formation

The private sector circulates knowledge within the healthcare industry to optimize diagnosis and treatment based on the latest research. Educating healthcare providers on international guidelines and standards is an effective way of improving patient care (Pfizer, 2020). The private sector also contributes to the development of standard protocols based on emerging research for NCD management to ultimately improve health-seeking behaviour and adherence (AB InBev, 2018; Abdool-Gaffar et al., 2011; Ahmed et al., 2015; AstraZeneca, 2021; Laar et al., 2019; Pati et al., 2020; Ratzan et al., 2013).

### Investment and finance

In the final review, Investment and Finance, eight themes were elucidated: cost of treatment; regulation; insurance; subsidization; direct investment; collaborative financing; innovative financing; research (Table 1).

#### Cost of treatment

The cost of NCD treatment and services in LMICs greatly impacts financing of NCD control and management through financial accessibility and protection. The private sector impacts this through the high (Anson et al., 2012; Ashigbie et al., 2020; Beran et al., 2019; Cameron et al., 2009; Khuluza & Haefele-Abah, 2019; Ladusingh et al., 2018; Lekshmi et al., 2014; Mendis et al., 2007; Rahman et al., 2013; Sado & Sufa, 2016; Singh et al., 2016; Subramanian et al., 2018; Tripathy & Prasad, 2018; Tusubira et al., 2020; van Mourik et al., 2010; World Bank, 2013) and variable (Armstrong-Hough et al., 2020) prices at which services and treatments are provided, as well as the extent of out-of-pocket (OOP) expenditure (Dugee et al., 2018; Ganju et al., 2020; Ladusingh et al., 2018; Rahman et al., 2013; Tripathy & Prasad, 2018; World Bank, 2013) at private facilities. LMICs in which prices did not vary considerably between public and private facilities had national price control strategies (Heidari et al., 2019). Pricing along the supply chain and manufacturing process (Cameron et al., 2009; FAO & UNICEF, 2020; Holt et al., 2017; Kanzler & Ng, 2012; Kishore et al., 2015; Mendis et al., 2007; van Mourik et al., 2010) and retail mark-ups (Cameron et al., 2009; Kishore et al., 2015; Mendis et al., 2007; Mhlanga & Suleman, 2014) further contribute to these prices, and negatively impact NCD financing. Addressing these excessive mark-ups through regulation strategies will help bring patient prices down (van Mourik et al., 2010).

#### Regulation

Regulation strategies involving the private sector impact the financing of NCD control and management by improving financial affordability and protection and generating revenue. Private sector self-regulation and adherence to guidelines limits prices (Cameron et al., 2009; Kanzler & Ng, 2012; You et al., 2019) while taxes on products generates revenue from the private sector while encouraging NCD-friendly changes (FAO & UNICEF, 2020; Mendis & Chestnov, 2013; The Advisory Group on the Governance of the Private Sector for UHC, 2020). It is important to consider, however, that limiting private sector prices could be a disincentive for stocking products and thus have an adverse effect on availability (Cameron et al., 2009).

#### Health insurance

Private health insurance is both a facilitator and barrier to accessing NCD care (Tusubira et al., 2020). It improves financial protection regarding NCD treatment and service (El-Saharty et al., 2013; Holt et al., 2017; Kanzler & Ng, 2012; Lambert & Kolbe-Alexander, 2013; Rahman et al., 2013) while relieving financial pressures on public health systems (Cattaneo & Piemonte, 2021; Dutta & Ly, 2018; Kanzler & Ng, 2012). Private insurance can also be expensive, however, which may be a barrier to affordable NCD care (El-Saharty et al., 2013; Kanzler & Ng, 2012; The Advisory Group on the Governance of the Private Sector for UHC, 2020). NCD and population ageing trends could impact private insurance company business models should they be required to pay out associated expensive insurance claims, thus these industries are interested in exploring risk management interventions (Prescott & Stibbe, 2017).

#### Subsidization

Subsidization of products and services by the private sector improves affordability of services and treatments that contribute to NCD mitigation (Lambert & Kolbe-Alexander, 2013; Prescott & Stibbe, 2017; World Bank, 2013). Improving the prices and providing incentives improves engagement and participation in NCD mitigation interventions (Lambert & Kolbe-Alexander, 2013; Prescott & Stibbe, 2017).

#### Direct investment

The private sector directly invests in NCD control and management programming to support interventions and purchase new equipment (Allen, 2017; El-Saharty et al., 2013). Overcoming barriers to private investment and leveraging private sector funds will unleash critical new flow of capital to catalyse NCD service delivery (Jones, 2021; Shellaby & Henshall, 2018; The Advisory Group on the Governance of the Private Sector for UHC, 2020) fill financing gaps for quality NCD care caused by limited domestic spending (El-Saharty et al., 2013; Jones, 2021; Shellaby & Henshall, 2018) curtail future health costs (Prescott & Stibbe, 2017) and offer more innovative products and solutions to NCDs (Bloom et al., 2011; Ganju et al., 2020; Jones, 2021). This can be done through corporate social responsibility initiatives (Jones, 2021; The Advisory Group on the Governance of the Private Sector for UHC, 2020) and investment in “best buys” (Bloom et al., 2011; Ganju et al., 2020; Jones, 2021; Shellaby & Henshall, 2018).

#### Collaborative financing

The private sector can collaborate with other sectors to mobilize funding for NCD initiatives, which aligns objectives and ensures the even distribution of funding (Allen, 2017; Ganju et al., 2020; Jones, 2021; Mendis & Chestnov, 2013; The Advisory Group on the Governance of the Private Sector for UHC, 2020; UNICEF, 2019; WHO, 2019). PPPs specifically leverage private and public investment to support NCD programming (Allen, 2017; Dutta & Ly, 2018; Jones, 2021; Shellaby & Henshall, 2018; UNICEF, 2019).

#### Innovative financing

Innovative financing mechanisms leverage private funding for NCD programming through a plethora of paths. These programs provide innovative incentives and platforms for funding, and reduce risk of investment for private partners to improve engagement and ultimately support for NCD mitigation programming (Allen, 2017; Jones, 2021; van Mourik et al., 2010; WHO, 2013, 2021). Programs include development bonds (Jones, 2021; Shellaby & Henshall, 2018) development bank lending (Jones, 2021; Shellaby & Henshall, 2018) and multi-donor trust funds (MDTFs) (UNICEF, 2019; WHO, 2019).

#### Research

Private sector development of new originator brand products protected by intellectual property rights impede attempts to improve medicine affordability, preventing largescale distribution of affordable generic options (Beran et al., 2019; Cameron et al., 2009; Heidari et al., 2019; Khuluza & Haefele-Abah, 2019; Kishore et al., 2015; Lekshmi et al., 2014; Mendis et al., 2007; Mhlanga & Suleman, 2014; van Mourik et al., 2010; You et al., 2019). Permitting generics to be produced and sold and engaging private sector generic manufacturers will lower prices of available NCD medicines (Kishore et al., 2015; van Mourik et al., 2010). The private sector also invests directly in research to support innovation and developments in NCD control and management (FAO & UNICEF, 2020; Prescott & Stibbe, 2017; Schmutz et al., 2019). It has been observed, however that publications sponsored exclusively by food and beverage companies are more likely to have conclusions favourable to the sponsoring company (Moodie et al., 2013).

### Quality assessment

#### PPPs

Five studies were considered high quality (Das et al., 2017; Kraak et al., 2012; Ota et al., 2018; Shannon et al., 2019; Thow, Verma, etal., 2018) nine medium quality (Alizadeh et al., 2020; Healthy Caribbean Coalition & NCD Alliance, 2017; Johnson et al., 2018; Jones, 2021; Moscetti & Taylor, 2015; Oluwole & Kraemer, 2013; Silva et al., 2017; UNICEF, 2020; WHO, 2016) and eleven low quality (Goroff & Reich, 2010; Hawkes & Buse, 2011; Hospedales & Jané-Llopis, 2011; Moodie et al., 2013; Prescott & Stibbe, 2017; Trager, 2020; UNICEF, 2019; WHO GCM/NCD Working Group, 2018; WHO, 2013, 2019; World Economic Forum & PAHO, 2013) (Appendix D). Studies were primarily downgraded for incomplete description of methods and data (Goroff & Reich, 2010; Hawkes & Buse, 2011; Hospedales & Jané-Llopis, 2011; Johnson et al., 2018; Moodie et al., 2013; Oluwole & Kraemer, 2013; Silva et al., 2017; Trager, 2020; UNICEF, 2019, 2020; WHO, 2013, 2016, 2019; World Economic Forum & PAHO, 2013) incomplete description of sampling (Alizadeh et al., 2020; Goroff & Reich, 2010; Hawkes & Buse, 2011; Moodie et al., 2013; Prescott & Stibbe, 2017; Trager, 2020; UNICEF, 2019; WHO, 2019) incomplete description of data analysis (Goroff & Reich, 2010; Hawkes & Buse, 2011; Healthy Caribbean Coalition & NCD Alliance, 2017; Hospedales & Jané-Llopis, 2011; Johnson et al., 2018; Jones, 2021; Kraak et al., 2012; Moodie et al., 2013; Oluwole & Kraemer, 2013; Ota et al., 2018; Prescott & Stibbe, 2017; Silva et al., 2017; Trager, 2020; UNICEF, 2019, 2020; WHO GCM/NCD Working Group, 2018; WHO, 2013, 2016, 2019; World Economic Forum & PAHO, 2013) and insufficient mention of ethics and bias (Goroff & Reich, 2010; Hawkes & Buse, 2011; Healthy Caribbean Coalition & NCD Alliance, 2017; Hospedales & Jané-Llopis, 2011; Johnson et al., 2018; Jones, 2021; Moodie et al., 2013; Moscetti & Taylor, 2015; Oluwole & Kraemer, 2013; Prescott & Stibbe, 2017; Silva et al., 2017; Trager, 2020; UNICEF, 2019, 2020; WHO GCM/NCD Working Group, 2018; WHO, 2013, 2016, 2019; World Economic Forum & PAHO, 2013).

#### Governance & policy

Seventeen studies were considered high quality (Bhojani et al., 2014; Buse et al., 2017; Carriedo et al., 2021; Cetthakrikul et al., 2019; Coriakula et al., 2018; Gómez, 2019; Jaichuen et al., 2018; Lauber et al., 2020; Mialon & Da Silva Gomes, 2019; Mialon et al., 2016, 2020, 2021; Oladepo et al., 2018; Sacks et al., 2015; Sanni et al., 2018; Suzuki et al., 2021; Thow, Greenberg, etal., 2018) five medium quality (Ben Romdhane et al., 2015; WHO, 2016, 2020, 2021; Williams, 2015) and eleven low quality (Bergman et al., 2012; Casswell, 2013, 2019; George, 2018; Gortmaker et al., 2012; Khayatzadeh-Mahani et al., 2018; Mariath & Martins, 2020; Moodie et al., 2013; Moscetti & Taylor, 2015; Myers et al., 2017; Tangcharoensathien et al., 2019) (Appendix D). Studies were primarily downgraded for insufficient mention of ethics and bias (Ben Romdhane et al., 2015; Bergman et al., 2012; Buse et al., 2017; Carriedo et al., 2021; Casswell, 2013, 2019; Cetthakrikul et al., 2019; Gortmaker et al., 2012; Gómez, 2019; Khayatzadeh-Mahani et al., 2018; Lauber et al., 2020; Mariath & Martins, 2020; Mialon & Da Silva Gomes, 2019; Moodie et al., 2013; Moscetti & Taylor, 2015; Myers et al., 2017; Sacks et al., 2015; Tangcharoensathien et al., 2019; Thow, Greenberg, et al., 2018; WHO, 2016, 2020, 2021; Williams, 2015) incomplete description of methods and data (Ben Romdhane et al., 2015; Bergman et al., 2012; Bhojani et al., 2014; Buse et al., 2017; Carriedo et al., 2021; Casswell, 2013, 2019; George, 2018; Gortmaker et al., 2012; Khayatzadeh-Mahani et al., 2018; Mariath & Martins, 2020; Mialon & Da Silva Gomes, 2019; Mialon et al., 2021; Moodie et al., 2013; Moscetti & Taylor, 2015; Myers et al., 2017; Tangcharoensathien et al., 2019; WHO, 2016, 2020, 2021; Williams, 2015) and incomplete description of data analysis (Ben Romdhane et al., 2015; Bergman et al., 2012; Buse et al., 2017; Casswell, 2013, 2019; George, 2018; Gortmaker et al., 2012; Khayatzadeh-Mahani et al., 2018; Mariath & Martins, 2020; Mialon & Da Silva Gomes, 2019; Mialon et al., 2021; Moodie et al., 2013; Moscetti & Taylor, 2015; Myers et al., 2017; Tangcharoensathien et al., 2019; WHO, 2016, 2020, 2021; Williams, 2015).

#### Healthcare provision

Nine studies were considered high quality, six medium quality, and seven low quality (Appendix D). Studies were primarily downgraded for insufficient mention of ethics and bias (Abraham et al., 2009; Agarwal, 2005; Ashigbie et al., 2020; Atre, 2015; Balasubramaniam et al., 2014; Bhojani et al., 2012; Bissell et al., 2016; Cataife, 2012; Dabare et al., 2014; Mukherjee et al., 2011; Pandian et al., 2007; Polanczyk & Ribeiro, 2009; Rawal et al., 2020, 2020; Subramanian et al., 2018; Syed et al., 2018; Wearne et al., 2019; Wirtz et al., 2018) and incomplete description of sampling (Abraham et al., 2009; Agarwal, 2005; Ashigbie et al., 2020; Atre, 2015; Balasubramaniam et al., 2014; Bhojani et al., 2012; Bissell et al., 2016; Cataife, 2012; Dabare et al., 2014; Pandian et al., 2007; Polanczyk & Ribeiro, 2009; Subramanian et al., 2018; Syed et al., 2018; Wearne et al., 2019).

#### Innovation

Five studies were considered high quality, three medium quality, and seven low quality (Appendix D). Studies were primarily downgraded for insufficient mention of ethics and bias (AstraZeneca, 2021; da Cruz Paula et al., 2020; Doshi et al., 2021; Evert et al., 2019; Medtronic Foundation n.d.; Grancelli, 2005; Hancock et al., 2011; IFC, 2011; Lambert & Kolbe-Alexander, 2013; Rockers et al., 2019; Saldarriaga et al., 2017; Shannon et al., 2019; Tzeel, 2011; U.S. Department of State, 2011; van de Vijver et al., 2013) and incomplete description of sampling (AstraZeneca, 2021; Doshi et al., 2021; Evert et al., 2019; Hancock et al., 2011; IFC, 2011; Lambert & Kolbe-Alexander, 2013; Saldarriaga et al., 2017; Shannon et al., 2019; Tzeel, 2011; U.S. Department of State, 2011; van de Vijver et al., 2013; Medtronic Foundation n.d.).

#### Knowledge educator

Four studies were considered high quality, three medium quality, and seven low quality (Appendix D). Studies were primarily downgraded for insufficient mention of ethics and bias (AB InBev, 2018; Abdool-Gaffar et al., 2011; AVON Global Scholars n.d.; AstraZeneca, 2021; Laar et al., 2019; Malekzadeh et al., 2020; Merck , 2021; Novo Nordisk, 2017; Pastakia et al., 2020; Patel et al., 2010; Pfizer, 2020; Ratzan et al., 2013; Reeves, 2011) and incomplete description of sampling (AB InBev, 2018; Abdool-Gaffar et al., 2011; AVON Global Scholars n.d.; Ahmed et al., 2015; AstraZeneca, 2021; Laar et al., 2019; Malekzadeh et al., 2020; Merck , 2021; Novo Nordisk, 2017; Pastakia et al., 2020; Pfizer, 2020; Ratzan et al., 2013; Reeves, 2011).

#### Investment and finance

Sixteen studies were considered high quality (Anson et al., 2012; Armstrong-Hough et al., 2020; Ashigbie et al., 2020; Cameron et al., 2009; Dugee et al., 2018; Heidari et al., 2019; Khuluza & Haefele-Abah, 2019; Mendis et al., 2007; Mhlanga & Suleman, 2014; Rahman et al., 2013; Sado & Sufa, 2016; Singh et al., 2016; Subramanian et al., 2018; Tripathy & Prasad, 2018; Tusubira et al., 2020; van Mourik et al., 2010) twelve medium quality (Allen, 2017; Bloom et al., 2011; FAO & UNICEF, 2020; Jones, 2021; Ladusingh et al., 2018; Lambert & Kolbe-Alexander, 2013; Lekshmi et al., 2014; Mendis & Chestnov, 2013; Schmutz et al., 2019; The Advisory Group on the Governance of the Private Sector for UHC, 2020; World Bank, 2013; You et al., 2019) and fourteen low quality Prescott and Stibbe (2017), WHO (2013), Institute for Health Metrics and Evaluation (IHME) (2020) (Cattaneo & Piemonte, 2021, 2021; Clarke & Paviza, 2018; Dutta & Ly, 2018; Ganju et al., 2020; Holt et al., 2017; Kanzler & Ng, 2012; Kishore et al., 2015; Moodie et al., 2013; Shellaby & Henshall, 2018; UNICEF, 2019) (Appendix D). Studies were mainly downgraded due to incomplete descriptions of data analysis (Allen, 2017; Beran et al., 2019; Cattaneo & Piemonte, 2021; Dutta & Ly, 2018; El-Saharty et al., 2013; FAO & UNICEF, 2020; Ganju et al., 2020; Jones, 2021; Kanzler & Ng, 2012; Kishore et al., 2015; Lambert & Kolbe-Alexander, 2013; Mendis & Chestnov, 2013; Moodie et al., 2013; Prescott & Stibbe, 2017; Shellaby & Henshall, 2018; The Advisory Group on the Governance of the Private Sector for UHC, 2020; UNICEF, 2019; WHO, 2013, 2019; World Bank, 2013) and incomplete descriptions of ethics and bias (Anson et al., 2012; Armstrong-Hough et al., 2020; Beran et al., 2019; Bloom et al., 2011; Cameron et al., 2009; Cattaneo & Piemonte, 2021; Dugee et al., 2018; Dutta & Ly, 2018; El-Saharty et al., 2013; FAO & UNICEF, 2020; Ganju et al., 2020; Heidari et al., 2019; Holt et al., 2017; Jones, 2021; Kanzler & Ng, 2012; Kishore et al., 2015; Ladusingh et al., 2018; Lekshmi et al., 2014; Mendis & Chestnov, 2013; Mendis et al., 2007; Moodie et al., 2013; Prescott & Stibbe, 2017; Schmutz et al., 2019; Shellaby & Henshall, 2018; The Advisory Group on the Governance of the Private Sector for UHC, 2020; UNICEF, 2019; van Mourik et al., 2010; WHO, 2013, 2019; World Bank, 2013; You et al., 2019).

## Discussion

The aim of this study was to elucidate the for-profit private sector’s roles in NCD prevention and management in LMICs through the six pillars outlined in our framework. A thematic synthesis approach allowed for the elucidation and categorization of these roles. These findings will be instrumental for LMICs considering engagement with the private sector, and who are facing an increased presence of these industries in the field of NCD prevention and management.

The private sector’s roles via PPPs involved: coordination (align goals, leverage expertise), financial resources, provision (medicine and service provision), health promotion (health education, physical activity, nutrition), capacity building (training), innovation (research, technology), and policy (policy influencer). There was a lack of examples of PPPs in LMICs with the private sector having a role in science, technology, and innovation (STI) research for NCD prevention and management. This suggests an area to consider for future PPPs. Many governments and the UN promote PPPs to combat NCDs, but the evidence of unhealthy commodity industry involvement delivering health benefits is lacking, as PPPs are sometimes used as delaying tactics to prevent stronger regulation (Jaichuen et al., 2018).

In terms of Governance and Policy, the themes included: lobbying (fight policy, legal strategies, policy influencer, government infiltration, collaboration), industry perception (framing, shape evidence, economic importance), and regulation (self-regulation, evade regulation). The private sector plays an influential role in the proliferation of NCD-related policies that impact LMICs. Industries often aim to create policy environments favouring their business model at the cost of NCD mitigation. With practices like lobbying becoming increasingly influential in middle-income countries (Williams, 2015) it is important to ensure regulation and transparency. The private sector holds substantial power in governance and policy, which could positively impact NCD control and management if industries are motivated to do so (i.e., incentives, clear shifts in public demand). Currently, evidence suggests that the private sector more detrimental to NCD-related governance and policy than beneficial.

The private sector’s roles in Healthcare Provision fell under the themes of diagnosis and treatment, infrastructure, and availability, accessibility, and affordability. In LMICs, due to limited supply of health products and services, individuals must utilize the private sector. The private sector often provides healthcare services that are missing in public healthcare, however, the care provided in the private sector is more expensive and patients must often decide between paying high OOP expenses or forgoing recommended treatment (Armstrong-Hough et al., 2020; Balasubramaniam et al., 2014; Bissell et al., 2016; Kasonde et al., 2019; Mhlanga & Suleman, 2014; Musinguzi et al., 2015; van de Vijver et al., 2013). Individuals opt to go to private facilities for NCD care due to perceived higher quality or because required services were not available in public centres (Atre, 2015; Rawal et al., 2020; Tusubira et al., 2020).

Regarding Innovation, the private sector’s roles fell under the themes product innovation (medications, HIT and ICT, technology, upgrade of existing technologies), process innovation (increasing outreach, private health insurance), marketing innovation (tailoring services), research, and innovation dissemination. Innovations by the private sector not only improve a process, product, or marketing, but also reach a new set of consumers in LMICs due to tailoring (Bhattacharyya et al., 2010; Parikh, 2013).

The roles of the private sector as knowledge educators in NCD prevention and management fell under the following themes: training (building research capacity, building health workforce capacity), health promotion (awareness, accessibility), and industry framework and guideline formation. The role in education transcends awareness and builds the capacity of available in-country resources to operate effectively. Awareness alone does not lead to behavioural changes (Pati et al., 2020).

Lastly, private sector roles in investment and finance included: cost of treatment (high prices, OOP expenditures, supply chain/manufacturer pricing, mark-ups), regulation, private insurance, subsidization, direct investment, collaborative financing (multi-sector collaboration, PPPs), innovative financing (innovative programs, development bonds, development bank lending, MDTFs), and research (originators vs. generics, funding). The private sector plays a role in NCD investment and financing that is both positive and negative, contributing to high costs and lack of affordability, while subsidizing some costs and providing capital to fund initiatives. The positive roles of the private sector appear to outnumber the negatives, and with adequate regulatory measures the private sector will be a constructive influence on NCD prevention and management in LMICs.

### Conflicts of interest

While the private sector fills many roles in NCD mitigation, their involvement is complex and prone to conflicts of interest (COIs), especially industries involved in unhealthy commodities (Silva et al., 2017). The commercial activities, strategies, and actions that the private sector uses to promote their products affect the health of populations and are termed commercial determinants of health (Kickbusch et al., 2016; World Health Organization, 2021). These determinants impact a diverse range of health outcomes that pertain to NCDs, such as obesity, hypertension, diabetes, cardiovascular health, and cancer, while also exacerbating pre-existing health disparities that exist (World Health Organization, 2021). LMICs are for example more vulnerable to the commercial determinants of health and face greater pressure from the private sector (World Health Organization, 2021). The venues through which the private sector enacts these commercial determinants of health align with those listed in this review, such as marketing, lobbying, social responsibility strategies, extensive supply chains, highlighting the COIs that persist with private sector involvement (Kickbusch et al., 2016).

PPPs involve shared decision-making powers amongst partners, allowing private partners to play a role in decisions concerning NCD agendas, goals, strategies, resources, and responsibilities (WHO, 2016). As a result, some governments, NGOs, researchers, and professionals are hesitant to enter partnerships with the private sector due to potential COIs (Hawkes & Buse, 2011). Governance (Goroff & Reich, 2010; Hospedales & Jané-Llopis, 2011) accountability mechanisms (WHO GCM/NCD Working Group, 2018) contracts (World Economic Forum & PAHO, 2013) organizational structures (Hawkes & Buse, 2011) transparency (Hawkes & Buse, 2011) and balance between interests (Hospedales & Jané-Llopis, 2011) are important to prevent a single partner, or group of aligned partners, from holding control or negatively interfering. The threat of government regulation and specified timelines can elicit meaningful commitments and help avoid COIs (Moodie et al., 2013; WHO GCM/NCD Working Group, 2018).

While private sector involvement in research has positive attributes, industry funding has biased studies in favour of the funding industry (Moscetti & Taylor, 2015). Furthermore, while research and efforts towards reformulation of unhealthy products benefit HICs where consumption is high, reformulation may encourage consumption in LMICs where consumption is lower (Moodie et al., 2013).

There is also a lack of evidence of the efficacy of self-regulation (Moodie et al., 2013; Myers et al., 2017) leaving doubts that it is an effective form of governance (Myers et al., 2017). For self-regulation to be effective, standards such as proportionate power distributions, and external, objective evaluation mechanisms are necessary (Buse et al., 2017). Experts believe that public regulation is the only effective and evidence-based approach for achieving positive changes in industry practices (Buse et al., 2017; Moodie et al., 2013).

Risks associated with private sector involvement are reduced should the private entities be those that are directly or indirectly part of the solution (i.e., sports manufacturers, urban architects, gyms, health insurance, and pharmaceutical companies) (Prescott & Stibbe, 2017).

### Strengths

This series of systematic reviews had many strengths. They were unique in their goal to investigate the role of the for-profit private sector through these six pillars specifically in NCD prevention and management in LMICs. The results thus fill a gap in the literature which will help better inform the development of health systems in LMICs. The search performed was also comprehensive and included 6 databases and the websites of over 15 different relevant organizations and companies. Furthermore, the databases searched included publications from a variety of disciplines, including biomedical sciences and business, enabling the collection of a diverse range of publications to support a holistic analysis.

### Weaknesses

Despite these strengths, this study included limitations. First, the screening, data extraction, and quality assessment for each review were completed by only one reviewer, which could introduce bias. To mitigate this, we ensured that a clear set of inclusion and exclusion criteria were utilized, and explicit instructions for data extraction and quality assessment were provided. Another limitation is that only studies available in English were included, which may have resulted in the automatic exclusion of relevant papers. Lastly, many studies included were determined to be of low quality, suggesting that the data used for the review could be subject to bias. These low ratings, however, could be attributed to the format of included studies and the lack of formal methods sections within them.

## Policy perspective

The findings of this review can be used to guide future policy decisions pertaining to the role of the private sector in NCD prevention and management in LMICs. Despite the prevalence and risk of COIs, it is possible to successfully use and work with the private sector to make meaningful changes in NCD burdens. In LMICs where public spending is often limited, the private sector can aid in the mobilization of capital and expertise to compensate. Policies should be considered that involve the private sector in the provision of NCD-related care, services, and resources that provides benefit to public health goals. This must be accompanied by frequent, ongoing evaluations of the value of this contribution and the impact on NCD burdens. These policies may involve incentives to reformulate products to be healthier, improve the distribution and price of health-promoting items (i.e., medication), provide high quality and affordable health services, and fund research that is unbiased and contributes to improved population health outcomes. In addition, there must also be strict, transparent regulatory frameworks that limit the power of the private sector in decision-making or other areas where they may have a negative influence. These frameworks should be under consistent monitoring to assess their effectiveness in mitigating potential negative effects of COIs.

## Conclusion

In conclusion, this review filled a gap in the literature and provided an overview of the roles that the private sector plays through PPPs, governance and policy, healthcare provision, knowledge educator, innovation, and investment and finance in NCD prevention and management in LMICs. Private sector involvement is prone to COIs, which must be considered throughout the planning and implementation of engagements to ensure optimal action against NCDs. Moving forward, the results of this study can provide guidance to the governments of LMICs regarding private sector involvement in NCD prevention and management.

## Supplementary Material

Supplemental Material

## Data Availability

Data relevant to the study are included in the article or uploaded as supplementary information in the Appendix. If further information is required, this can be provided by corresponding author on reasonable request.
