# Supplementary material for: The role of the private sector in noncommunicable disease prevention and management in low- and middle-income countries: a series of systematic reviews and thematic syntheses
Source: Int J Qual Stud Health Well-being. 2023 Jan 2;18(1):2156099. doi: 10.1080/17482631.2022.2156099 (PMC9815432; doi:10.1080/17482631.2022.2156099)
Supplement: Supplemental Material [file ZQHW_A_2156099_SM9690.docx]

**Appendices:**

Appendix A: Embase Search Strategies

Appendix B: Data Extraction Form

Appendix C: Study Characteristics Tables

Appendix D: Quality Assessment

**Appendix A:** Embase Search Strategies

*PPP Systematic Review*

|  | **Subject heading** | **Text words** |
| --- | --- | --- |
| Private sector | ‘Private sector’ /exp | ‘Private sector*’:ti,ab,kw  ‘Private’:ti,ab,kw |
| Noncommunicable disease | ‘Chronic disease’ /exp  ‘Non communicable disease’ /exp | ‘Chronic disease*’:ti,ab,kw  ‘NCD*’:ti,ab,kw  ‘Noncommunicable disease*’:ti,ab,kw  ‘Non-communicable disease*’:ti,ab,kw |
| PPPs | ‘Public-private partnership’/exp | ‘Public-private partnership*’:ti,ab,kw  ‘Private-public partnership*’:ti,ab,kw  ‘Private-public collaboration*’:ti,ab,kw  ‘Public-private collaboration*’:ti,ab,kw  ‘Private-public cooperation*’:ti,ab,kw  ‘Public-private cooperation*’:ti,ab,kw  ‘Public-private mix*’:ti,ab,kw  ‘Public-private sector partnership*’:ti,ab,kw  ‘PPP*’:ti,ab,kw |
| Search for: (private sector) **AND** (noncommunicable disease) **AND** (PPPs)  Filters: publication year: 2000-2021, English only | | |

*Governance and Policy Systematic Review*

|  | **Subject heading** | **Text words** |
| --- | --- | --- |
| Private sector | ‘Private sector’ /exp | ‘Private sector*’:ti,ab,kw  ‘Private’:ti,ab,kw |
| Noncommunicable disease | ‘Chronic disease’ /exp  ‘Non communicable disease’ /exp | ‘Chronic disease*’:ti,ab,kw  ‘NCD*’:ti,ab,kw  ‘Noncommunicable disease*’:ti,ab,kw  ‘Non-communicable disease*’:ti,ab,kw |
| Governance & policy | ‘Health care policy’ /exp  ‘Public policy’ /exp  ‘Policy’/ exp | ‘Health care polic*’:ti,ab,kw  ‘Healthcare polic*’:ti,ab,kw  ‘Health polic*’:ti,ab,kw  ‘Public polic*’:ti,ab,kw  ‘Policy’:ti,ab,kw  ‘Policies’:ti,ab,kw  ‘Governance’:ti,ab,kw  ‘Regulation*’:ti,ab,kw  ‘Regulatory’:ti,ab,kw  ‘Law*’:ti,ab,kw |
| Search for: (private sector) **AND** (noncommunicable disease) **AND** (governance and policy)  Filters: publication year: 2000-2021, English only | | |

*Healthcare Provision Systematic Review*

|  | **Subject heading** | **Text words** |
| --- | --- | --- |
| Private sector | ‘Private sector’ /exp | ‘Private sector*’:ti,ab,kw  ‘Private’:ti,ab,kw |
| Noncommunicable disease | ‘Chronic disease’ /exp  ‘Non communicable disease’ /exp | ‘Chronic disease*’:ti,ab,kw  ‘NCD*’:ti,ab,kw  ‘Noncommunicable disease*’:ti,ab,kw  ‘Non-communicable disease*’:ti,ab,kw |
| Healthcare provision | ‘Health care delivery’/exp  ‘Health care personnel’/exp  ‘Health care system’/exp | ‘Educat*’:ti,ab,kw  ‘Training’:ti,ab,kw  ‘Knowledge’:ti,ab,kw  ‘Behavior* change’:ti,ab,kw  ‘Behaviour* change’:ti,ab,kw  ‘Scholarship*’:ti,ab,kw |
| Search for: (private sector) **AND** (noncommunicable disease) **AND** (healthcare provision)  Filters: publication year: 2000-2021, English only | | |

*Knowledge Educator Systematic Review*

|  | **Subject heading** | **Text words** |
| --- | --- | --- |
| Private sector | ‘Private sector’ /exp | ‘Private sector*’:ti,ab,kw  ‘Private’:ti,ab,kw |
| Noncommunicable disease | ‘Chronic disease’ /exp  ‘Non communicable disease’ /exp | ‘Chronic disease*’:ti,ab,kw  ‘NCD*’:ti,ab,kw  ‘Noncommunicable disease*’:ti,ab,kw  ‘Non-communicable disease*’:ti,ab,kw |
| Knowledge educator | ‘Education’/exp  ‘Clinical education’/exp  ‘Health education’/exp  ‘Medical education’/exp  ‘Behavior change’/exp  ‘Professional development’/exp | ‘Health care delivery’:ti,ab,kw  ‘Healthcare delivery’:ti,ab,kw  ‘Health care personnel’:ti,ab,kw  ‘Healthcare personnel’:ti,ab,kw  ‘Health care system’:ti,ab,kw  ‘Healthcare system’:ti,ab,kw  ‘Health care provision’:ti,ab,kw  ‘Healthcare provision’:ti,ab,kw  ‘Health care supply’:ti,ab,kw  ‘Healthcare supply’:ti,ab,kw  ‘Delivery of health care’:ti,ab,kw  ‘Delivery of healthcare’:ti,ab,kw |
| Search for: (private sector) **AND** (noncommunicable disease) **AND** (knowledge educator)  Filters: publication year: 2000-2021, English only | | |

*Innovation Systematic Review*

|  | **Subject heading** | **Text words** |
| --- | --- | --- |
| Private sector | ‘Private sector’ /exp | ‘Private sector*’:ti,ab,kw  ‘Private’:ti,ab,kw |
| Noncommunicable disease | ‘Chronic disease’ /exp  ‘Non communicable disease’ /exp | ‘Chronic disease*’:ti,ab,kw  ‘NCD*’:ti,ab,kw  ‘Noncommunicable disease*’:ti,ab,kw  ‘Non-communicable disease*’:ti,ab,kw |
| Innovation | ‘Research’ /exp  ‘Medical research’ /exp  ‘Technology’ /exp  ‘Medical technology’ /exp  ‘Product development’ /exp | ‘Innovati*’:ti,ab,kw  ‘Technolog*’:ti,ab,kw  ‘Tech’:ti,ab,kw  ‘Biotech*’:ti,ab,kw  ‘Research*’:ti,ab,kw |
| Search for: (private sector) **AND** (noncommunicable disease) **AND** (innovation)  Filters: publication year: 2000-2021, English only | | |

*Investment and Finance Systematic Review*

|  | **Subject heading** | **Text words** |
| --- | --- | --- |
| Private sector | ‘Private sector’ /exp | ‘Private sector*’:ti,ab,kw  ‘Private’:ti,ab,kw |
| Chronic disease | ‘Chronic disease’ /exp  ‘Non communicable disease’ /exp | ‘Chronic disease*’:ti,ab,kw  ‘NCD*’:ti,ab,kw  ‘Noncommunicable disease*’:ti,ab,kw  ‘Non-communicable disease*’:ti,ab,kw |
| Investment & finance | ‘Investment’/exp  ‘Economic development’/exp  ‘Financial management’/exp  ‘Health care financing’/exp  ‘Health insurance’/exp  “Health care cost”/exp | ‘Invest*’:ti,ab,kw  ‘Economic development’:ti,ab,kw  ‘Financ*’:ti,ab,kw  ‘Financial management’:ti,ab,kw  ‘Health care financing’:ti,ab,kw  ‘Healthcare financing’:ti,ab,kw  ‘Health insurance’:ti,ab,kw  ‘Insurance’:ti,ab,kw  ‘Health care cost*’:ti,ab,kw  ‘Healthcare cost*’:ti,ab,kw |
| Search for: (private sector) **AND** (NCD) **AND** (investment & finance)  Filters: publication year: 2000-2021, English only | | |

**Appendix B:** Data Extraction Form

| **Main Category** | **Subcategory** | **Description** |
| --- | --- | --- |
| **Basic information:** | Author |  |
|  | Title |  |
|  | Journal |  |
|  | Funder(s) |  |
|  | Year of Publication |  |
| **Context & participants:** | Participants | Characteristics of the participants |
|  | Context | Context in which the study was embedded (i.e., where issue of interest emerged, setting of study sites, etc.) |
| **Study focus & methods** | Aim/objectives |  |
|  | Research questions |  |
|  | Study design | Methods used by the study (for qualitative studies, for example, interview, focus groups, observations, document analysis, etc.) |
|  | Sampling approach | The way the sample was selected, size of sample, etc. |
|  | Data collection methods |  |
|  | Data analysis approach |  |
|  | Theoretical model (if applicable) |  |
| **Findings:** | Example of **PPPs/Governance & Policy/Healthcare Provision/Knowledge Educator/Innovation/Investment and Finance** |  |
|  | Roles of **PPPs/Governance & Policy/Healthcare Provision/Knowledge Educator/Innovation/Investment and Finance** |  |
|  | Additional findings (if applicable) |  |
|  | Conclusions |  |
|  | Discussion or opinions | What the author(s) argues |
|  | Implications for practice/policy with respect to **PPPs/Governance & Policy/Healthcare Provision/Knowledge Educator/Innovation/Investment and Finance** |  |
|  | Study strengths & limitations |  |

**Appendix C:** Study Characteristics Tables

*PPP Systematic Review*

| **Author/Organization:** | **Title:** | **Study design:** | **Context:** | **Key findings:** |
| --- | --- | --- | --- | --- |
| Alizadeh et al., 2020 | Preventive Community-Based Strategies of Cardiovascular Diseases in Iran: A Multi-Method Study | Review followed by focus group discussion and questionnaires | Iran (upper-middle income) has increasing prevalence of NCD risk factors, partially due to aging society | PPPs are effective in rendering preventive health services and are efficient in special economic conditions  PPPs can provide quality services at low costs  Increasing PPP financial support may be effective in reducing CVD  Private sector help build infrastructure via financial donations in partnerships with government |
| Das et al., 2017 | Generic versus branded medicines: An observational study among patients with chronic diseases attending a public hospital outpatient department | Cross-sectional analysis using questionnaires | Suburban Kolkata in West Bengal, India (lower-middle income) has initiated FPMS PPP in government hospitals to combat chronic disease burden and negative attitudes towards generic drugs | Fair Price Medicine Shop (FPMS) is run through PPP where government provides space/infrastructure and private partner undertakes procurement/dispensing activities of generic drugs under mutually agreed upon contract  FPMS supplies drugs at substantial discounts and maintains mandatory list of items to be stocked for supply to hospital patients  FPMS has improved perceived effectiveness, safety and adherence to generic drugs |
| Goroff & Reich, 2010 | Partnerships to provide care and medicine for chronic diseases: A model for emerging markets | Review | Emerging markets and LMICs have high NCD burden that’s expected to increase with availability and use of NCD drugs uncertain, inconsistent, ineffective, and financially crippling | Chronic Disease Partnership Model recommends pharmaceutical manufacturer helps establish support enterprises in emerging markets to provide comprehensive, integrated, high-quality care for specific chronic diseases via partnership with local health providers  Differential pricing agreement negotiated through PPP where pharmaceutical manufacturer supplies drugs to partnership at prices and terms negotiated between them and partnership that are lower than in HIC; direct supply to partnership also mitigates distribution channel costs  Partnership evaluates and monitors generic medicine quality and saves costs by procuring high-quality low-cost options and provides as part of package |
| Hawkes & Buse, 2011 | Public health sector and food industry interaction: It’s time to clarify the term ‘partnership’ and be honest about underlying interests | Viewpoint | Globally, governments looking to promote healthy eating at population level to combat concerns about unhealthy diets, obesity and chronic diseases; food industry interested in being part of solution via PPPs | PPPs allow to more effectively achieve shared goals than working alone, and reach consensus about needed action through pooled expertise, ideas, skills and resources  PPPs are platforms for discussion amongst partners and can reach broader range of populations  Partnerships are mix of structured interactions involving range of activities with shared decision-making power among partners  Many governments, NGOs, researchers, professionals hesitant to enter PPP due to COIs  Good governance, transparency of private interests needed to ensure public health objectives come first |
| Health Caribbean Coalition (HCC) & NCD Alliance, 2017 | Preventing Childhood Obesity in the Caribbean | Report | Caribbean region (HICs, LMICs) faces NCD developmental burden, and increasing obesity rates with children becoming obese at younger ages | NCD Alliance program “Expanding Access to Care, Supporting Global, Regional and Country Level NCD Action” includes partnership with Medtronic Philanthropy, providing financial and technical resources to HCC in support of civil society’s contributions to NCD prevention and control in Caribbean  Engage partners, including private sector, to develop and implement strategies to advocate for, promote, organize and contribute to interventions enabling safe physical activity, recreation  Partner with private sector to mobilize resources and develop strategies to combat obesity |
| Hospedales & Jane-Llopis, 2011 | A multistakeholder platform to promote health and prevent noncommunicable diseases in the region of the Americas: The Pan American Health Organization Partners Forum for Action | Narrative report | Region of the Americas has highest NCD and obesity burden  Many risk factors require cross-sectoral approach but lack global/regional mechanism to ensure optimized multi-stakeholder NCD response | PAHO Partners Forum for Action on Chronic Disease involves partners from private sector, member states, civil society & focuses on 4 main NCDs and risks; ensures supportive governance and resource mechanisms to address needs, offering training/tools for effective health-related multistakeholder partnership creation and delivery  PPPs provide platform for raising awareness, dialogue, joint action among government, civil society, private sector to prevent NCDs and promote health  Support and strengthen existing partnerships in countries and collaborate within PPP to identify opportunities for innovation and further engagement  Successful partnerships need balance between interests, clear governance, staff with appropriate skillsets, shared values, and clarity of objectives |
| Johnson et al., 2018 | Global partnerships to support noncommunicable disease care in low and middle-income countries: Lessons from HIV/AIDS | Review | LMICs have emerging NCD pandemic with features that mirror early stages of HIV crisis, thus opportunities exist to leverage existing HIV partnership chronic care models for NCDs | PRRR involves private partners like Merck, GSK, Bristol-Myers Squibb, that expands availability of cervical and breast cancer services, especially for high-risk HIV+ women  Merck provided >265,000 GARDASIL donations via PRRR  PPPs contribute service delivery, financial/technology support, healthy systems capacity support, implementation research to improve delivery  Through PPPs private sector contributes technical skills like marketing, sales, product design, product distribution, supply chain, ICT, provider training  Local partnerships can fill gaps by uniting government, academic and community interests |
| Jones, 2021 | Global action on financing cervical cancer elimination: Funding secondary prevention services in low resource settings | Review and in-depth interviews | Cervical cancer is 4^th^ most commonly detected cancer in women; women in LMICs disproportionately affected by cervical cancer morbidity & mortality | PRRR leverages public and private investment to combat cervical and breast cancers, ran in 5 sub-Saharan African countries along with Peru  PRRR focused on education, vaccination, screening and increasing access to treatment and training for healthcare providers |
| Kraak et al., 2011 | Balancing the benefits and risks of public-private partnerships to address the global double burden of malnutrition | Review | Resource-constrained health systems face tremendous pressure from double burden of malnutrition and overweight, obesity and NCDs, thus new stakeholder engagement and governance structures such as PPPs are recommended | Philanthropic partnerships involve limited engagement, with reciprocity and activities peripherally important to partners’ missions  Transactional partnerships involve higher levels of interaction and resource investment; build mutually beneficial relationships to advance each partner’s agenda via compatibility among organizational values, missions, goals  Transformational partnerships involve highest level of engagement, resource investment, managerial complexity and relationships to mutually influence institutional cultures and practices of partners  IFBA is transformational partnership involving 8 food and beverage companies in response to rising obesity rates  PPPs can raise visibility of nutrition and health on policy agendas; mobilize funds and advocate for research; strengthen food-system processes and delivery systems; facilitate technology transfer; expand access to medications, vaccines, healthy food and beverages  Challenge to balance private commercial interests and public health interests thus need systematic and transparent approaches  Effective PPPs develop trusting relationships to share information, technology and promote innovation; leverage financial or in-kind resources, expertise, networks, distribution systems |
| Moodie et al., 2013 | Profits and pandemics: Prevention of harmful effects of tobacco, alcohol, and ultra-processed food and drink industries | Narrative report | LMICs are seeing rise in sales of unhealthy commodities which are major drivers of NCD epidemics, but UN High-Level Meeting on NCDs called for multisectoral action including private sector and potentially unhealthy commodity industries | Belief that association with industry leads to greater success than acting independently  Partnerships can lead to reformulation of foods and beverages  Little objective evidence that PPPs deliver health benefits, and are not just delaying tactics  HIC markets saturated with ultra-processed products so benefit from reformulation, but benefits in LIC less obvious and danger apparent if consumption increases following reformulation and targeting by UHC industry  Definitive outcomes of PPPs must be independently and objectively monitored to establish efficacy  Precautionary principle argues against PPPs as no evidence partnership with alcohol and ultra-processed food/beverage industries is safe or effective unless driven by threat of government regulation |
| Moscetti & Taylor, 2015 | Take Me to Your Liter: Politics, Power, and Public-Private Partnerships with the Sugar-Sweetened Beverage Industry in the Post-2015 Development Agenda | Narrative report including case studies | NCDs are major threat to health and development, and many leaders suggest for PPPs with food and beverage industries  >60% obese people live in developing countries and economic costs of such NCDs unprecedented which may threaten productivity of workforce and economic development | IFBA is collaboration between food and beverage companies that pledges to reformulate products to be healthier and restrict unhealthy food marketing  NCD Roundtable is coalition with partnership between NGOs, universities, think tanks, private sector, and other civil society organizations which aims to raise profile of NCDs through policy dialogue and engagement  NCD Roundtable gave industries façade of legitimacy and allowed them to participate in HLMs and other international meetings not open to private sector  PAHO Partners Forum for Action on NCDs (PAFNCD) involves PPP with government, NGO, academic and private sector members  Food and beverage industry is welcome PAFNCD partner and financer, with numerous large donations from partners like Coca-Cola, Nestle and Unilever in exchange for influence  PPPS overcome resource constraints via resource sharing amongst partners  PPPs channel private funding into research, but may bias findings in favour of industry |
| Oluwole & Kraemer, 2013 | Innovative public–private partnership: a diagonal approach to combating women’s cancers in Africa | Narrative report | LMICs have poor breast and cervical cancer prognoses partly due to treatment barriers, which PRRR PPP aims to address in Botswana, Zambia, and some coverage in Cote d’Ivoire, Ethiopia, Kenya, Mozambique, Nigeria, Rwanda, South Africa, Uganda, and Tanzania | PRRR innovative partnership leverages public and private investments and builds on PEPFAR to combat breast and cervical cancer in sub-Saharan Africa and Latin America  PRRR PPP partners include: George W Bush Institute, US PEPFAR, Susan G Komen for the Cure, and Joint United Nations Programme on HIV/AIDS, as well as Becton, Dickinson and Company, Bill & Melinda Gates Foundation, Bristol-Myers Squibb Foundation, Caris Foundation, GlaxoSmithKline (GSK), IBM, Merck, QIAGEN, and other organizations with more limited engagement  PRRR focuses on improving access to HPV vaccine, increasing awareness of breast and cervical cancer prevention, diagnosis and treatment, reducing stigma around cancer diagnosis  PRRR fills gaps in national cancer control strategies identified and prioritized by governments, making it country-led and focused on country-set priorities  IBM supports health management information systems  >27,049 women in Zambia screened for cervical cancer through PRRR programs in 14-month period  >40 health workers in Zambia trained “see and treat” cervical cancer screening and treatment approach  PRRR members pledged USD >85 million in financial and in-kind support for first 5 years of activity  Private organizations offer expertise that others cannot match and can respond nimbly to country’s needs |
| Ota et al., 2018 | Proceedings of the first African Health Forum: effective partnerships and intersectoral collaborations are critical for attainment of Universal Health Coverage in Africa | Narrative report based on Forum | African countries face rising NCD burden so are working towards universal healthcare, and called first-ever WHO Africa Health Forum | PPPs can facilitate common interest in building healthier societies by advocating for universal healthcare and leveraging expertise |
| Prescott & Stibbe, 2017 | Better Together: Unleashing the Power of the Private Sector to Tackle Non-Communicable Diseases | Report | Scale of NCD epidemic means need to put as many resources towards problem as possible with all-of-society approach with private sector, in hopes that collaboration brings wider range of resources | Lower risk of COI in partnerships if are with companies that are part of solution to problem  Be He@lthy Be Mobile PPP for NCD prevention uses mobile technology to help combat growing NCD burden; partners include WHO, ITU, NCD Alliance, Bupa, Novartis, GSK, Sanofi, Asian Development Bank, African Development Bank, Verizon Wireless, IFPMA  PPP has toolkits covering issues like diabetes prevention, smoking cessation, cervical cancer diagnosis  PPP allowed for innovation through technology to support holistic approach to prevention  Within partnership, companies can apply technical, research, commercial, legal, financial, marketing, facilitation, training and project management skills  Combining resources in partnership allows for greater outputs than working separately  Gaining profit does not represent COI; need to be aware of commercial interests |
| Shannon et al., 2019 | Innovating to increase access to diabetes care in Kenya: an evaluation of Novo Nordisk’s base of the pyramid project | Interviews, questionnaires, focus group discussions, site visits, and document reviews from various stakeholders | Kenya (lower-middle income) prevalence of diabetes predicted to rise, with many undiagnosed, placing burden on strained healthcare system as many in LMICs struggle to receive basic diabetes care | BoP PPP aims to facilitate access to diabetes care for working poor in LMICs, and was initiated by Novo Nordisk in 28/47 Kenyan counties  Partners include Kenyan Ministry of Health, county Government Departments of Health, national and local drug distribution networks (Philips Pharmaceuticals Ltd., MEDS), faith-based organizations, Kenya Defeat Diabetes Association, and Novo Nordisk  BoP brings together stakeholders to ensure 5 pillars: increased awareness of diabetes, early diagnosis of diabetes, access to quality care by trained healthcare professionals, stable and affordable insulin supply, and improved self-management through patient education  BoP Programme activities include establish centers of excellence for diabetes care at local public hospitals, free screening and awareness campaigns, development of patient education materials in local languages, training of healthcare professionals, pharmacists, nutritionists in diabetes prevention and care  Novo Nordisk signed memorandum as part of PPP that details every link of distribution chain to make it more difficult for distributors and actors to exceed agreed upon prices for insulin, limiting mark-ups while retaining incentives for regular supply |
| Silva et al., 2017 | Can the risk in public-private partnerships be classified? | Review | Globally, PPPs will play increasingly relevant role in NCD control, but COIs very possible in many countries, such as Brazil (upper-middle income) | THE PEP coordinated by WHO & adopted successfully in HIC, MIC, LMIC and LIC promoted beneficial health & economic effects of cycling/walking via PPP with public health, epidemiology, transportation, and economics specialists  PPPs ideally promote health with products or consumption of items that don’t cause harm  Public agencies can benefit from private sector collaboration in areas where they lack specialization (i.e., development of research & technologies)  Possible partnerships are with sports, unprocessed foods; complex to partner with UHC industries so need safeguards and established commitments |
| Thow et al., 2018 | Improving policy coherence for food security and nutrition in South Africa: a qualitative policy analysis | Qualitative policy analysis using interviews based on policy theory | India (lower-middle income) is facing double burden of malnutrition and NCDs, and has favourable climate to grow fruits and vegetables yet majority exported and not domestically consumed | PPPs are opportunity to engage stakeholders strategically to benefit from diverse expertise across supply chain to improve factors like storage and transport  PPP opportunity to use commodity-based organizations (krishi vidya Kendras, KVKs) run by government to connect farmers to private sector buyers  Thailand urban supply of fruits and vegetables improved by PPP between supermarkets and public sector, which streamlined urban supply chains via central distribution center, scaled up certification schemes, and training for producers, wholesalers and retailers |
| Trager, 2020 | A Better Approach to Fighting Chronic Diseases | Narrative report | NCDs are responsible for 71% of global deaths prior to COVID-19 pandemic and will continue to be problem once pandemic ends, leaving a large economic burden and thus requiring payment solutions such as PPPs | PPPs designed to rebuild Lesotho’s Queen Mamohato Memorial Hospital in Maseru, South Africa (upper-middle income) with partnership between Lesotho Ministry of Health and South African firm, Netcare, but significant COI emerged as to whether private sector operator would be incentivized to cut corners and compromise quality of care provided  COI mitigated by developing sophisticated payment schedule with highly specific performance metrics  PPPs mean sharing of risks, resources and decision-making authority  PPPs can spur innovation by leveraging operational capacity of both public and private sectors  Cash-strapped ministries can reduce risk and ensure funds go further by managing allocation of risks and opportunities more effectively via PPPs |
| UNICEF, 2019 | Programme Guidance for Early Life Prevention of Non-Communicable Diseases | Report | Majority of premature NCD deaths occur in LMICs, and engagement of multiple sectors needed to reduce societal, environmental, and behavioural NCD risks | Private sector is source of partnership and funding for raising profile of NCD risk among constituency  Partnership with commercial sector can give business data and expertise to generate evidence, while leveraging business assets, technology, communications and reach  PPPs free of COIs are needed to raise resources for NCD interventions |
| UNICEF, 2020 | Nutrition, for every child: UNICEF Nutrition Strategy 2020-2030 | Report | LMICs face unsolved burden of child malnutrition with simultaneous rise in overweight and obesity prevalence, increasingly affected poorer households | Public-private multi-donor partners can leverage financial resources  Strategic partnerships with private sector are critical lever to allow UNICEF to share responsibilities, optimize resources and maximize results  Engage strategically with private sector to advocate for business policies, practices and products that support optimal nutrition for all children, adolescents and women |
| WHO, 2013 | Global Action Plan for the Prevention and Control of Noncommunicable Diseases 2013-2020 | Report | LMICs bear 86% of burden of premature NCD deaths, leading to cumulative economic losses of US$ 7 trillion between 2011-2025 | Build community capacity in promoting healthy diets and lifestyles through collaborative partnerships with private sector  Multisectoral partnerships promote cooperation at all levels among governmental agencies, intergovernmental organizations, NGOs, civil society and private sector to strengthen NCD prevention/control efforts |
| WHO, 2016 | SHAKE the salt habit: The SHAKE technical package for salt reduction | Report | Raised BP is leading factor for global disease burden, specifically in LMICs where risk of death from high BP and CVD is more than double that of HIC, this is important to reduce salt intake | PPPs provide opportunity to engage with and use expertise and interests of diverse stakeholders that are not directly involved in program leadership, such as food industry |
| WHO GCM/NCD Working group, 2018 | Engaging with the private sector for the prevention and control of NCDs | Report | Heads of State and Government acknowledged NCDs as major challenge for development, and called on private sector to help make progress | Governments should actively explore opportunities through PPPs to increase access to safe, effective, affordable and quality-assured essential NCD medicines and health technologies to support achievement of targets of Global Action Plan on NCDs and contribute to UHC  Need to safeguard public health interests from undue influence of real or perceived or potential COIs through strong regulatory frameworks underpinned by legislation, transparent monitoring  Governments should better align private sector incentives with national public health goals to encourage and facilitate stronger contribution to NCD prevention and control |
| WHO, 2019 | It’s time to walk the talk: WHO independent high-level commission on noncommunicable diseases final report | Report | Many countries lagging in implementing NCD strategies, with limited progress made to secure effective and meaningful commitments, contributions and actions from private sector | Align private sector action with government policy to promote public health outcomes for NCDs through how they produce, market, advertise and sell products  Recommend WHO to increase engagement with private sector through various approaches, including PPPs, to promote private sector’s effective and meaningful contributions to global NCD targets and goals, and to provide technical support to Member States to increase capacity |
| World Economic Forum & PAHO, 2013 | Multistakeholder Collaboration for Healthy - Living Toolkit for Joint Action | Report | Decades of socioeconomic gains threatened by NCDs, so need multistakeholder collaboration involving private sector to comprehensively promote healthy living and have sustained impact on NCD levels | PPPs can be source of financing  Leverage core competencies of each partner, expertise, logistics and implementation capacity  Private sector on executive boards through PPPs can help dictate progress of programs, provide insight on how to improve  Discovery Private Insurance’s Vitality Program involves partnerships with civil society and public agencies (Department of Education) and is consumer-focused, incentive-based health insurance program that provides low-cost access to gyms, significant discounts on foods and rewards including discounts on consumer products for practicing healthy behaviours  Agita São Paulo, Brazil is international multi-stakeholder collaboration with Executive Board involving over 150 institutions from education, sports, health, industry, commerce and services; uses mass media campaign to increase population levels of physical activity using promotional materials that were culturally adapted to Brazilian values and mega-events |

*Governance and Policy Systematic Review*

| **Author/Organization:** | **Title:** | **Study design:** | **Context:** | **Key findings:** |
| --- | --- | --- | --- | --- |
| Ben Romdhane et al., 2015 | Health system challenges of NCDs in Tunisia | Document analysis with semi-structured interviews and case studies | Tunisia (lower-middle income) is undergoing rapid demographic and epidemiological change with NCDs now exceeding communicable diseases  Lack of research on healthy system and policy environment | Multiple actors involved in NCD prevention and control programs, including private health providers, pharmaceutical industry, mass media  Tunisian pharmaceutical industry has grown considerably, becoming more influential interest group (some fear influence has encouraged private sector physicians to ignore guidelines and norms proposed by Ministry of Health) |
| Bergman et al., 2012 | Diabetes prevention: Global health policy and perspectives from the ground | Narrative report | 50-80% of individuals in LMICs with diabetes are diagnosed with potential for developing chronic complications  70% diabetes cases in LMICs result from consumption of high-calorie foods and physical inactivity | Realistic policy interventions must be directed at making healthy choices easier  Strong industry collaboration has made significant progress on several key areas like reformulating to reduce sugar, innovating smaller package sizes, labeling calories and sugar, restricting marketing to children, withdrawing full-calorie sodas from schools, investment in activity programs |
| Bhojani et al., 2014 | Health System Challenges in Organizing Quality Diabetes Care for Urban Poor in South India | Cross-sectional study with semi-structured interviews and thematic analysis | Leading causes of death and disabilities in India (lower-middle income) are chronic conditions  India has 2^nd^ highest number of people living with diabetes in the world, behind China | Pharmaceutical companies had easy access to doctors for influencing practice through periodic personal visits by company reps, sponsoring of continuing medical activities, and provision of medical literature to doctors  Common for private doctors to bribe lower-level government officials to get necessary license/registration for their facilities or to avoid punitive actions |
| Buse et al., 2017 | Healthy people and healthy profits? Elaborating a conceptual framework for governing the commercial determinants of non-communicable diseases and identifying options for reducing risk exposure | Review | Growing NCD burden in LMICs and increasing risk of exposure are major impediments to 2030 SDGs  Grappling with commercial drivers of ill-health requires addressing profit-driven epidemics characterising some NCDs which are rising in LMICs | IFBA formed with focus on tackling NCDs via initiatives like reformulation, responsible marketing  PepsiCo, Coca-Cola contributed to policy recommendations for UN 2011 HLM on NCDs via participation in NCDRT and sponsored side-events  Industry self-regulation popular with commercial sector, voluntary, involves limited public expenditure, may generate better compliance  Big Food keen to demonstrate active response to NCD epidemic via public voluntary codes of conduct and pledges  Co-regulation through partnership raises concerns for industry potential to wield influence over global health policy  Alliances between tobacco and alcohol industries in 3 areas: taxation, legal regulation, and advertising/marketing restrictions |
| Carriedo et al., 2021 | The political economy of sugar-sweetened beverage taxation in Latin America: lessons from Mexico, Chile and Colombia | Document analysis with case studies | Excess SSB consumption associated with diet-related NCDs and taxing has become increasingly supported policy intervention  5 Latin American countries enacted SSB legislation between 2014 and 2018 but large TNCs cloud public policy debate | Constituency building: relationships with key opinion leaders and policymakers in community and health organizations cultivated  Strategic efforts of TNCs to influence policy decisions documented for food, beverage, alcohol and tobacco industries  TNCs (particularly SSB producers) have engaged in countries’ social and poverty alleviation programs often via PPPs or CSR  TNCs and national SSB producers opposed measures of SSB tax in Mexico, represented by several business chambers  SSB tax in Mexico enacted in 2014 partially due to poor industry self-regulation  Intense industry lobbying and harassment of activists reported in Colombia  SSB industry won lawsuit and uses CSR in Colombia  In Mexico and Colombia corporate interests influenced media |
| Casswell, 2013 | Vested interests in addiction research and policy Why do we not see the corporate interests of the alcohol industry as clearly as we see those of the tobacco industry? | Review | Adoption of Political Declaration on NCDs by UN provided insight into current politics of health  At UN HLM on NCDs, it was clear how differently alcohol and tobacco were perceived | Alcohol producer strategy to establish credibility as ‘partners’ in efforts to address expansion of alcohol-related harm  In addition to direct lobbying, producers and front organizations engaged in stakeholder marketing to portray alcohol as part of solution  ICAP active in countries with large, youthful populations, growing economies  In Africa, ICAP collaborated with SAB Miller in co-hosting stakeholder meetings to develop alcohol policy, with focus on largely ineffective strategies  Subsequent alcohol policy in Malawi carried out without industry interference documented influence exerted by alcohol industry  ICAP also co-hosted national policy development workshops in Vietnam, Papua New Guinea |
| Casswell, 2019 | Addressing NCDs: Penetration of the Producers of Hazardous Products into Global Health Environment Requires a Strong Response Comment on ‘Addressing NCDs: Challenges From Industry Market Promotion and Interferences’ | Commentary | Comment on editorial which warned about challenges of NCDs from 4 industries: tobacco, alcohol, food, and breast milk substitute | For TNCs to maximize profits, especially by expansion into middle-income countries, must avoid implementation of effective policies limiting over-supply, marketing and affordability of products  Diageo has reported to shareholders success of their measures gaining access to policy-making process  Industries influence policy by framing themselves as part of solution, supported by CSR activities  GHC's NCDRT: PepsiCo and Coca-Cola contributed to policy recommendations to 2011 UN HLM on NCDs, sponsored side events  2018 recommendations from UN HLM judged as having been very compromised by input from countries representing interests of commercial determinants |
| Cetthakrikul et al., 2019 | Assessment of the stated policies of prominent food companies related to obesity and non-communicable disease (NCD) prevention in Thailand | Policy analysis | Obesity and diet-related NCD burdens are affecting countries globally, increasingly developing countries like Thailand (upper-middle income). Thai food environment has changed dramatically with greater supply and demand of unhealthy products. SSB tax was introduced in 2017 | 4 companies had product reformulation policies, but most were non-specific in nature without quantitative targets  3 companies had policies for food marketing to children, but didn’t specify detail beyond that this applied to saturated fats, trans-fatty acids, free sugars, and salt  12 companies had policies for provision of nutritional information but not all stated this complied with relevant labelling restrictions; some companies went above commitments to comply with regulations  3 companies had stated policies and commitments on availability and affordability of healthier products; referred to developing company investment plans to improve delivery and access for children and rural populations; did not define level of price that is affordable or what is meant by healthy product |
| Coriakula et al., 2018 | The development and implementation of a new import duty on palm oil to reduce non-communicable disease in Fiji | Qualitative approach using case studies | NCDs place significant health burden on Pacific Island countries, including Fiji (upper-middle income). Policy interventions to curb NCDs in Fiji include 32% increase in import duty on palm oil. | Only obstacles to implementation included counter-lobbying from traders, retailers, importers  Food industry against palm oil duty so wrote to Minister  Changed labels to vegetable oil to avoid duties |
| George, 2018 | An Unwelcome Seat at the Table: The Role of Big Food in Public and Private Standard-Setting and its Implications for NCD Regulation | Narrative report | Political Declaration of UN HLM on NCDs included call for private sector involvement. SDGs recognize need for multi-stakeholder approach. Sustainable development requires private sector involvement. | IFBA/food industry played influential role in UN civil society hearings that shaped 2011 UN Political Declaration on NCDs  6 tactics used by tobacco industry to undermine policies: hijack political and legislative process; exaggerate economic importance of industry; manipulate public opinion to gain respectability; fabricating support through front groups; discrediting proven science; intimidating governments with litigation or threat of; use normal scientific uncertainty to discredit evidence  Food companies use legal, regulatory, and societal mechanisms to protect promotion of their products like lobbying for favourable laws, regulations, and trade agreements; arrangements with food and nutrition experts to obtain evidence; personal connections via sponsorships, educational activities; public relations; legal action against unfavourable regulation  All 5 Mexican delegates at 2011 session of Codex Committee on Nutrition and Foods for Special Dietary Uses represented private sector  PAFNCD included IFBA representatives, allowing them to participate in developing PAFNCD strategic policies  2009 Fiji initiative allowing food industry to work in government policy resulted in heavy lobbying and no changes in SSB marketing or availability |
| Gómez 2019 | Coca-Cola’s political and policy influence in Mexico: Understanding the role of institutions, interests and divided society | Case study with stakeholder interviews | Obesity and diabetes epidemics in Mexico (upper-middle income) triggered partly by SSB industry.Government has responded through variety of NCD policies. Major industries like Coca-Cola have obstructed prioritization of these policies and have considerable influence. | Soda tax in 2008 and 2012 never adopted due to influence of sugary-beverage and fast-food industries in Mexico  Efforts in 2010 to prevent sale of soft drinks and other sweetened beverages in school blocked after vehement opposition from soda industry  SSB tax adopted in 2013, but future efforts to increase tax in 2016 unsuccessful  Coca-Cola worked with researchers, foundations, think-tanks, and sponsored conferences to downplay harm of increased sugar consumption, questioning scientific evidence behind these claims and policy effectiveness  Coca-Cola lobbyists and supportive labour unions had access to congressional members and committee meetings where they lobbied against soda taxes  Former President Vincente Fox was Coca-Cola executive and benefited from campaign donations and advice from corporate friends  Academics seeking funding have often supported Coca-Cola, other soda industries, and ConMexico foundation due to latter’s willingness to finance research and facilitate publication  Use media to question relationship between sodas and NCDs |
| Gortmaker et al., 2012 | Changing the Future of Obesity: Science, Policy, and Action | Narrative report | Steady increase in obesity prevalence globally including in most LMICs. Need action by government and relevant institutions but lack consensus on effective policy/programmatic strategies. | Most powerful activities by private sector relevant to public policy are lobbying activities which often undermine policies aimed to reduce obesity (i.e., regulating marketing to children, traffic light labelling, taxes on unhealthy food) |
| Jaichuen et al., 2018 | Corporate political activity of major food companies in Thailand: An assessment and policy recommendations | Policy review with semi-structured interviews | Food industry can influence individual and population level consumption behaviours, shape public preferences, and interfere in government policy. Thailand (upper-middle income) has third largest market of snack foods and non-alcoholic beverages in South-East Asia region. | Food industry prevented introduction of traffic light food labeling system  Types of CPA identified are information and messaging, financial incentives strategy, constituency building strategy (most common), legal strategies  Industry frequently referred to its economic importance in discussion with government and media; this framing is barrier to public health efforts for NCDs  Industry promotes deregulation, especially sugar companies stating SSB taxes would negatively impact farmers and employment  Many food industry sectors apply strategy of framing  Most frequently used techniques to shape evidence were fund research, pay experts to speak or promote evidence in favour of company, use data favouring company to promote public image, sponsor educational activities  Use financial incentives through donations to policymakers and political parties  Establish relationships with key opinion leaders, and with community  Public-private interactions with informal and formal relationships between policymakers and private sector, revolving door  Establish relationships with media agencies, journalists, other bloggers, to facilitate media advocacy in favour of industry; includes paying journalists  Use legal action by posing legal threats against public policy  Develop and promote alternatives to policies via policy substitution, voluntary agreements, self-regulation  Opposition fragmentation and destabilization strategy |
| Khayatzadeh-Mahani et al., 2018 | Could the WHO’s Framework on Engagement with Non-State Actors (FENSA) be a threat to tackling childhood obesity? | Commentary | Growth rate of childhood obesity 30% higher in LMICs vs. HICs. Majority of 41 million obese and overweight children under 5 years live in LMICs | Role of private sector in wider WHO policy process reinforced by FENSA, which encourages private sector involvement in WHO policy reformulation  Growing concern that corporate influence and lobbying at WHO likely to weaken regulatory and policy efforts targeting childhood obesity |
| Lauber et al., 2020 | Non-communicable disease governance in the era of the sustainable development goals: A qualitative analysis of food industry framing in WHO consultations | Frame analysis and policy review | UN’s shift to multi-stakeholder governance via SDGs invites many actors, including private sector, to policymaking table | FIA and IFBA highlight self-regulation as “cost-effective”, “measurable”, “flexible”, and able to “quickly respond to societal concerns”  Partnership framing and use of language around ‘multistakeholder’ and ‘multisectoral’ approaches also used to position food industry as legitimate policy actor with right to participate  GMA stated salt reduction strategies could not have been achieved without constructive engagement between health authorities and industry  Respondents framed regulation as inappropriate or unnecessary, arguing existing measures were sufficient, current voluntary actions achieved positive results, and non-statutory, self- or co-regulation would be preferable |
| Mariath & Martins, 2020 | Ultra-processed products industry operating as an interest group | Review | Participation of UPP industry in efforts to reduce obesity and diet-related NCDs has been questioned, due to evidence of policymaking interference. In Brazil (upper-middle income) industry participation mainly occurs with industry associations but hasn’t been established whether interests prevail in decision-making process. | Brazil private sector didn’t hide position contrary to regulation and preference for maintaining status quo  Lobbying is most usual activity of interest groups in pluralistic systems, in which several organizations seek to influence decision-making public policy  Lobbying includes information collection, preparation of policy drafts, definition of strategies for defense, search for allies, exercise of pressure  Evident interaction of political action of corporate interest groups to influence results of public policy intensified after publication of regulation  Privileged access of lobbyists plus economic power of industry facilitated softer version of regulation  Participation of private sector occurred in indirect manner via AVIA and ABIR  Empirical evidence of intense participation of ultra-processed industry in regulatory processes involving nutrition labeling and withdrawing of tax benefits on industrialized products |
| Mialon et al., 2016 | Analysis of the corporate political activity of major food industry actors in Fiji | Document analysis with semi-structured interviews | NCDs are leading cause of mortality in Fiji (upper-middle income). Some food products processed and sold by food industry are major contributors to NCD epidemic. Food industry widely identified as having strong economic and political power | Types of CPA identified are information messaging strategy, financial incentives strategy, constituency building strategy, legal strategies, policy substitution strategy  Food industry representatives make direct contact with policymakers to push agenda via lobbying  Most influential practice is framing as economic issue, stressing number of jobs supported and money generated for economy  Food industry framed debate on diet- and public health-related issues in Fiji in way favourable to industry, emphasizing role of physical activity, individuals, and parents vs. unhealthy food environments  Food industry, specifically Nestle, supplied educational materials to schools and communities (Kana Vinaka program)  Soft drink industry cherry picks evidence to share  Financial incentives like funding political parties influences on policies  Fijian government collaborating with major food industry actors to prevent and control NCDs; sees relationships as strength to public health, not threat  Journalists invited to events sponsored by food industry, building relationships  Food industry threatens legal action to prevent harmful publicity  Some food companies voluntarily reformulated some products, but don’t mention specific criteria to evaluate and monitor  Policy substitution employed to prevent implementation of new regulations |
| Mialon & Gomes, 2019 | Public health and the ultra-processed food and drink products industry: corporate political activity of major transnationals in Latin America and the Caribbean | Document analysis | UPP consumption has been linked to increased risk NCDs. Regulatory measures to control and prevent NCDs yet to be developed and implemented in many countries partially due to UPP industry influence. | Types of CPA identified are information messaging strategy (most common), constituency building strategy, policy substitution strategy  UPP industry actors lobbied governments and tried to influence decisions when health regulations discussed in Costa Rica (upper-middle income), Ecuador (upper-middle income), Guatemala (upper-middle income), Uruguay (high-income)  UPP industry actors stressed their economic importance in countries in which they operate like Argentina (upper-middle income), Brazil (upper-middle income), Colombia (upper-middle income), Ecuador (upper-middle income), Mexico (upper-middle income), Peru (upper-middle income), Trinidad and Tobago (high-income), Uruguay (high-income)  UPP industry actors framed debate in ways favourable to companies  All industry actors in study were members of IFBA and therefore pledged to adopt IFBA commitments regarding responsible marketing  UPP industry tried to shape evidence on diet- and public health-related issues  UPP industry tried to build relationships with health professionals, universities, communities, and policymakers  Evidence of policy substitution strategy for many countries, including Jamaica |
| Mialon et al., 2020 | ‘the architecture of the state was transformed in favour of the interests of companies’: Corporate political activity of the food industry in Colombia | Document analysis with informant interviews | Public health policies to improve food environments in Colombia (upper-middle income) are currently under development in response to increased NCD burden. Opposition to policies by food industry currently delaying and weakening efforts | Media capture: Ardila Lulle Group owns leading TV channel and beverage company Postobon, leading to cases of censorship of public health in Colombia  Nutresa has its own research centre on NCDs called Vidarium that disseminates information to children, parents, and teachers  Coca-Cola organized series of talks aimed at government entities to provide info on energy balance and adequate hydration, contributing to healthy lifestyles  ILSI Nor-Andino is industry front group that’s been criticized for its influence on science and policy in numerous countries  Influence of food industry on science could translate into political influence  Food industry tried to shape evidence in Colombia for SSB taxation by hiring two people to do unpublished, non-peer-reviewed studies against SSB taxation  Industry lobbies by co-opting new members of parliament, entering Congress  Industries donate to political campaigns, give in-kind gifts  Self-regulation favoured by industry and supported by government, adopted to avoid SSB taxes and series of proposals for state regulation  Food industry promoted providing nutrition information, conscious advertising, responsible marketing, reformulation, promoting healthy lifestyles |
| Mialon et al., 2021 | ‘I had never seen so many lobbyists’: Food industry political practices during the development of a new nutrition front-of-pack labelling system in Colombia | Document analysis with stakeholder interviews | Evidence that food industry CPA is one of principle obstacles to developing public health in Colombia (upper-middle income). Bill promoted in Colombia in 2018 involving FOPL, WL, and restricted marketing | Types of CPA identified are coalition management, information management, direct involvement and influence in policy, legal strategies, discursive strategies  Build alliances in communities, media, and health organizations through public-private initiatives  Food industry influences dissemination of evidence in ways favourable to industry by criticizing and suppressing evidence that doesn’t fit industry’s interests, hiring experts  Directly influence policy, including through lobbying of politicians or donations to political parties and policymakers, advocate for self-regulation  Food industry actors and their allies in government threatened legal actions during public hearings for FOPL in 2019  Food industry actors made numerous argument-based, discursive strategies when opposing WL system |
| Moodie et al., 2013 | Profits and pandemics: Prevention of harmful effects of tobacco, alcohol, and ultra-processed food and drink industries | Narrative report | LMICs are seeing rise in sales of unhealthy commodities and these industries are major drivers of NCD epidemics globally, but UN High-Level Meeting on NCDs called for multisectoral action including private sector and potentially unhealthy commodity industries but there is debate concerning the extent of potential COIs | Bias research findings (i.e., Philip Morris International Whitecoat project hiring doctors to publish ghostwritten confounder studies)  Co-opt policymakers and health professionals (i.e., SAB Miller and IPAC assisted Lesotho, Malawi, Uganda, and Botswana governments write alcohol control policies)  Lobby politicians and public officials to oppose public regulation (i.e., tobacco TNCs lobby policymakers and fund campaigns of those that support tobacco)  Encourage voters to oppose public health regulation (i.e., tobacco industry campaigns against taxation/regulation, deflect criticism by promoting areas outside of expertise)  Industry-operated, voluntary self-regulation is default approach of many governments and UN, preferred approach of industry  Conditional engagement: PPP supporting round-the-table interaction with industry to promote evidence-based policy  No evidence of effectiveness/safety of self-regulation and/or PPPs |
| Moscetti & Taylor, 2015 | Take Me to Your Liter: Politics, Power, and Public-Private Partnerships with the Sugar-Sweetened Beverage Industry in the Post-2015 Development Agenda | Narrative report with case studies | NCDs are global public health crisis and foreign policy priority. Many experts, governments, global leaders have rallied for whole-of-society approach. Almost ¾ NCD deaths and most premature deaths occur in LMICs. NCD risk factors on the rise in many LMICs | IFBA: collaboration between food and beverage companies with voluntary commitments to improve products and public health; pledged to reformulate products, improve nutrition information, restrict marketing to children  Industry-funded studies more likely to present conclusion of no positive association between SSB and obesity  NCDRT launched by GHC as coalition of civil society organizations including private sector to raise profile of NCDs through policy dialogue  NCDRT gave private sector equal voice in generating consensus policy recommendations, gave them high degree of legitimacy  PAHO partnered with World Economic Forum to establish PAFNCD which allowed corporations like Pfizer, Medtronic, and Coca-Cola to take lead role  PAFNCD viewed industries as partners and stakeholders in NCD policymaking  PAHO received substantial funding from private sector via PAFNCD |
| Myers et al., 2017 | Sugar and health in South Africa: Potential challenges to leveraging policy change | Narrative report | Excessive sugar consumption is major health concern and has led to sharp increases in obesity and NCDs. NCDs accounted for 40% deaths in South Africa (upper-middle income) in 2013. SSB tax would be effective in reducing obesity and associated NCDs. | Industry lobbied for protectionist measures out of concern for cheap sugar exports from Brazil and India, and in Mexico, bottlers and food companies launched media campaign against SSB tax proposed by President in 2013  2014 application by industry for increased import tariff granted by DTI, but met with objections from retailers, importers, Ministry of Trade, and Industry  South African sugar industry expected to offer significant resistance to tax  South African Sugar Association website has claims distorting or contradicting current scientific evidence, serves to confuse public  No regulatory action for sugar aside from self-regulatory measures by industry  Industry usually proposes self-regulation as most appropriate solution, but lack evidence for effectiveness  PPPs may be way to avoid harsher restrictions/regulations |
| Oladepo et al., 2018 | Analysis of tobacco control policies in Nigeria: historical development and application of multi-sectoral action | Policy document analysis with case study and key informant interviews | Tobacco use is major NCD risk factor and 3.7% Nigerians currently smoke tobacco. WHO “best buy” interventions for tobacco control include tax increases, smoke-free workplaces and public places, advertising bans, mass media campaigns. | BATN actively sought to halt tobacco bill passage and prevent from advancing to committee stage, and sponsored full-page advert to undermine anti-tobacco NGOs  Tobacco Bill eventually passed by House of National Assembly and Senate but not signed by President due to further pressure from tobacco industry and objection by some ministries  Tobacco industry involved in formulation of 2014 Standard for Tobacco and Tobacco Products, contrary to FCTC recommendations  10-year gap between Nigeria’s ratification of FCTC and formulation and passage of comprehensive tobacco bill, largely attributed to opposition and lobbying of tobacco industry |
| Sacks et al., 2015 | Comparison of food industry policies and commitments on marketing to children and product (re)formulation in Australia, New Zealand and Fiji. | Policy comparison | Unhealthy food environments are major drivers of obesity- and diet-related NCDs. Private sector organizations shape food environments. Governments have generally relied on industry self-regulation to reduce food marketing to children. Concern that companies make improvements in HICs but don’t apply them to LMICs | Lower proportion of manufacturers and restaurants in Fiji (upper-middle income) had fewer available policies or commitments compared to Australia and New Zealand |
| Sanni et al., 2018 | Assessment of the multi-sectoral approach to tobacco control policies in South Africa and Togo | Policy investigation with informant interviews | Tobacco is most important risk factor for NCDs and world’s largest cause of preventable illness and death. LMICs like South Africa (upper-middle income) and Togo (low-income) disproportionately impacted by NCDs | Actors from public sector, private sector, and civil society involved in policymaking on tobacco control in Togo and South Africa  Government had support from civil society to overcome private sector barriers  Low political context for tobacco in Togo so policymakers able to overcome resistance from representatives from tobacco and hospitality industries  High political context for tobacco in South Africa with stakeholders who have vested interests in blocking/weakening tobacco control policies |
| Suzuki et al., 2021 | Competing Frames in Global Health Governance: An Analysis of Stakeholder Influence on the Political Declaration on Non-communicable Diseases | Policy document analysis | NCDs are increasingly recognized as threat to health and development globally, and UN Political Declaration of HLM on NCDs in 2018. Declaration included consultations with Member States, intergovernmental organizations (IGOs), non-state actors like NGOs and private sector | Framing is important strategy to influence policy debates  Support for whole-of-society approach specifically inclusive of PPPS mentioned mainly by private sector, IGOs, Member States  Importance of regulations on harmful products strongly represented by NGOs and academic institutions but lesser endorsement from Member States, IGOs, private sector  ‘Healthy lifestyle’ promoted in 23 statements endorsing changing individual behaviours, awareness raising, public education, and physical activity; mainly represented by Member States, NGOs, private sector  Generally, NGOs, academic institutions and LMICs pursue ‘stricter’ form of governance of NCD risk factors, while the private sector and high-income countries oppose greater restrictions and promote whole-of-society approach |
| Tangcharoensathien et al., 2019 | Addressing NCDs: Challenges From Industry Market Promotion and Interferences | Editorial | Addressing NCD determinants is challenged by aggressive market promotion by tobacco, alcohol, and unhealthy food industries in emerging countries with fast economic development. Industry interference in government policies aimed at containing consumption of unhealthy products | Tobacco industry applies legal threats and files lawsuits against governments  Petition to Kenyan high court against proposed tobacco regulations stated 2% tax on tobacco products is arbitrary and capricious  Uganda tobacco industry also petitioned against Tobacco Control Act saying it unjustifiably singles out tobacco industry  Tobacco industry uses counteracting messages or other tactics  Alcohol industry highly effective and well-organized in gaining access to policymaking process, and building close relationships with policy actors  Alcohol industry casts doubt on scientific evidence, promoting weak survey-based evidence and making unsubstantiated claims  ICAP counters WHO recommendations and refutes evidence on most effective strategies to prevent alcohol-related harm  When Thailand introduced SSB tax in 2017, Government faced serious resistance from soft drink industry; Thai Beverage Industry Association questioned link between obesity and drinking SSBs |
| Thow et al., 2018 | Improving policy coherence for food security and nutrition in South Africa: a qualitative policy analysis | Document analysis with in-depth interviews | South Africa (upper-middle income) must address rising burden of diet-related chronic disease. Supply-side policy interventions critical to address double burden. Policy incoherence can occur between government action to promote healthy food supply and objectives for economic liberalization | Economic growth coalition focuses on role of economic growth and employment in delivering improved food security and nutrition outcomes in forums convened to inform policymaking  Food industry access to government, participate in range of forums and roundtables  Shift towards processed foods with negative nutrition implications framed as result of individual preferences for fat, salt, and sugar due to palatability  Solutions for NCDs framed as addressing personal factors via education  Promote belief that industry was key stakeholder in achieving food policy goals; portrayed as knowledgeable, avenue through which policy objective achieved  Industry and industry associations positioned themselves as key resources to achieve economic objectives and policy goals  Resources available to industry to fight policy much more significant |
| Williams, 2015 | The incursion of ‘Big Food’ in middle-income countries: a qualitative documentary case study analysis of the soft drinks industry in China and India | Document review with case study | Coca-Cola has invested $4 billion in China (upper-middle income) and plans to invest $5 billion in India (lower-middle income) by 2020. Soft drinks significantly associated with rising overweight, obesity, and diabetes globally and specifically in LMICs like China and India. | Coca-Cola, PepsiCo formed IBA, tasked with lobbying government on issues of taxation, industry guidelines and regulation, and defending against allegations  Soft drink industry lobbied against soda taxes in India, inviting political figures to sponsored events related to CSR initiatives, but has been largely unsuccessful  IBA argued that industry is major supplier of jobs, and that its growth will benefit millions of farmers across country  Ongoing uncertainty over restructuring tax system in India provides unique opportunity for stakeholders like Big Food, public health agencies, to try and influence government debates on taxation  Corporate lobbying becoming increasingly influential in countries like China, where is still relatively new practice |
| WHO, 2016 | SHAKE the salt habit: The SHAKE technical package for salt reduction | Report | Raised BP is leading factor for global disease burden, specifically in LMICs where risk of death from high BP and CVD is more than double that of HIC, this is important to reduce salt intake. | South African (upper-middle income) National Department of Health passed new mandatory sodium limits for processed foods in 2013 via extensive collaboration between government, academia, and industry  Argentina (upper-middle income) Ministry of Health established MENOS Sal MAS Vida campaign with aim of reducing population salt intakes  Voluntary agreement with local bakers to minimize sodium, leading to 18% reduction in salt content of bread between 2009-2010 |
| WHO, 2020 | Countdown To 2023: WHO report on global trans-fat elimination 2020 | Report | TFA intake associated with increased heart attacks and death from CHD. Replacing industrially produced TFA with healthier oils and fats is cost-effective and feasible, without changing tastes. May 2018, WHO called for global elimination of industrially produced TFA by 2023 and released REPLACE action framework. | IFBA member companies committed to not exceeding 2g of industrially produced TFA per 100g oils and fats in products globally by 2023  Nigerian Government (lower-middle income) drafted, announced, and notified for public comment a best-practice TFA policy in January 2020. Support from local industry reinforced NAFDAC’s commitment to implementing REPLACE action package  Peru (upper-middle income) has had mandatory limits capping TFA content at 2% in vegetable oils and fats, and 5% in other processed foods since 2018  Industry initially opposed regulations and tried to delay implementation, but civil society helped move process forward  Thailand (upper-middle income) implemented PHO ban January 2019 following stakeholder meetings with researchers, food manufacturers, and importers to determine best policy approach  Thai FDA held public hearings after making tentative policy decisions where consumers, food producers, importers, academia, and other government agencies demonstrated broad support for PHO ban |
| WHO, 2021 | WHO technical manual on tobacco tax policy and administration | Report | Tobacco taxation not only revenue source, but effective public health intervention to reduce tobacco consumption and its associated harms. Cigarettes have become more, rather than less, affordable in many LMICs over past decade | Industry tactics used to undermine tax increases include stockpiling; changing certain product characteristics; choosing time or price increase announcement strategically; adopting price-discriminating strategies or price-related promotions; using brand proliferation and price segmentation; differential shifting of tax increases across different price segments depending on market circumstances; lobbying government to distort interventions  In efforts to oppose tobacco tax increases, tobacco industry utilizes SCARE tactics (Smuggling and elicit trade; Court and legal challenges; Anti-poor rhetoric; Revenue reduction; Employment impact) to influence political economy of tobacco  Tobacco industry frames tobacco taxes as economic, not public health, issue |

*Healthcare Provision Systematic Review*

| **Author/Organization** | **Title:** | **Study type** | **Context** | **Key findings** |
| --- | --- | --- | --- | --- |
| Bissell et al., 2016 | Access to essential medicines to treat chronic respiratory disease in low-income countries | Quantitative and exploratory study | Despite measures put in place by the United Nations to address availability and affordability of essential medicines, essential medications for chronic respiratory diseases (CRD) are unavailable and unaffordable for many in low-income countries. | Availability of CRD essential medications was higher in the private sector than in the public sector  Essential medications for CRD cost much higher in private facilities than in public facilities  If medicine were not available in the public sector, individuals need to purchase at higher prices in the private sector. Sometimes, the medications were not available at all |
| Balasubramaniam et al., 2014 | A national survey of price and affordability of key essential medicines for children in Sri Lanka | Cross-sectional descriptive survey | Children in Sri Lanka (lower-middle income) are usually excluded from free medications offered by the government due lack of availability. | Child treatment in Sri Lanka for acute illnesses were affordable but treatments for chronic diseases were largely unaffordable  The lack of availability of essential medications in the public sector means individuals purchasing from the private sector for higher process, excluding majority of the population from receiving care  Private sector had more availability of the essential medications but access is limited due to cost |
| Dabare et al., 2014 | A national survey on availability, price and affordability of selected essential medicines for non communicable diseases in Sri Lanka | Quantitative study using survey | Low affordability and availability of medicines affect significant proportion of global population, including Sri Lanka (lower-middle income) | Selected essential medications for NCDs in Sri Lanka were found to be both affordable and available in both private and public facilities |
| Syed et al., 2018 | Access to Antihypertensive Medicines at the Household Level: A Study From 8 Counties of Kenya | Quantitative study using surveys | Of all WHO regions, Africa has highest prevalence of hypertension. Only 1 in 5 people diagnosed with hypertension in Kenya (lower-middle income) were taking medications | Majority of people survey received their diagnosis in the public sector but they most often purchased their medications from the private pharmacy  Besides long wait times to purchase medications from the public sector, people purchased from private sector because of low stock in the public sector  Public sector diagnoses diseases but the private sector is the “gatekeeper” to buying drugs in Kenya  Affordability is an important barrier to HTN treatment, including medicines |
| Wirtz et al., 2018 | Access to medicines for asthma, diabetes and hypertension in eight counties of Kenya | Quantitative study | The mortality rates in Kenya (lower-middle income) are increasing due to NCDs. There has been progress in delivery of care, but major gaps remain in availability and access to NCD care | About half of the respondents were diagnosed of hypertension and asthma in the public facility  Most diabetics were diagnosed in the private sector  Most respondents purchased treatment from the private sector |
| Bigdeli et al., 2016 | Access to Treatment for Diabetes and Hypertension in Rural Cambodia: Performance of Existing Social Health Protection Schemes | Mixed study with survey and interviews | NCD medications are on national essential medicines list. To protect the people from financial hardship in Cambodia (lower-middle income) due to NCD treatment, effective social health plans are being called for | Cambodia’s healthcare is dominated by the private sector and it is the preferred choice of treatment  Majority of the respondents were diagnosed in the private sector. Majority of this sought care in the private sector |
| Tusubira et al., 2020 | Accessing medicines for non-communicable diseases: Patients and health care workers’ experiences at public and private health facilities in Uganda | Qualitative descriptive study | NCDs are on the rise on LMICs. Availability of NCD medicines is generally low but not much is known regarding what affects accessibility to medications in Uganda (low-income) | Stocking of medications and supplies were low in public facilities  Private sector offered a wide range of NCD treatment services  Provision of NCD care is mostly in the public sector in Uganda  Private facilities provided care everyday and patient can access care when they wanted |
| Atre, 2015 | Addressing policy needs for prevention and control of type 2 diabetes in India | Narrative | India (lower-middle income) is home to 1/5 of global burden of diabetes. The government has initiated a program for prevention and control of diabetes | Majority of the people have to seek care diabetic care in the private sector  The private sector predominantly provides diabetes care in India |
| Ashigbie et al., 2020 | Availability and prices of medicines for non-communicable diseases at health facilities and retail drug outlets in Kenya: A cross-sectional survey in eight counties | Cross-sectional study | In Kenya (lower-middle income), NCDs are the leading cause of death | Medication availability was highest in the private sector  Kenyan do not have a reliable NCD treatment access particularly in the public sector |
| Musinguzi et al., 2015 | Capacity of Health Facilities to Manage Hypertension in Mukono and Buikwe Districts in Uganda: Challenges and Recommendations | Cross-sectional study | NCDs are a growing burden in LMICs. But the healthcare system of Uganda (low-income) is not equipped to manage chronic diseases | The availability of anti-hypertensive medications was high in the private sector  Some medications were only in stock in the private sector  Lack of medications and diagnostics equipment led people to seek care on the private sector |
| Bhojani et al., 2012 | Challenges in organizing quality diabetes care for the urban poor: a local health system perspective | Qualitative study | Unfavourable social determinants of heath are affecting access to healthcare by the poor | The private sector has more doctors, laboratories, hospitals, and pharmacies in the studies area.  The public sector had challenges with human resources, medicines, laboratories  Majority of diabetes patients in the area studies received care in the private sector |
| Agarwal, 2005 | Chronic kidney disease and its prevention in India. | Literature review | Chronic kidney disease is an NCD affecting India (lower-middle income) but due to non-existent CKD registry, the magnitude is unknown | Majority of the infrastructure including experts for dialysis management is in the private sector  It is much more expensive to received dialysis in the private sector than in the public sector |
| Rawal et al., 2020 | Community health workers for non-communicable disease prevention and control in Nepal: a qualitative study | Literature review and qualitative study using interviews | The burden of NCDs in Nepal (lower-middle income) requires strengthening of primary healthcare | Lack of regular supply in the public system  High availability of essential medications in the private sector |
| Angwenyi et al., 2020 | Context matters: A qualitative study of the practicalities and dilemmas of delivering integrated chronic care within primary and secondary care settings in a rural Malawian district | Qualitative study | Increasing burden of NCDs in Sub-Saharan Africa, specifically Malawi (low-income) requires re- strategizing of the delivery of care | Over one-third of health services is provided by the private sector  Resources for screening and diagnostic limited in public PHC facilities  Cardiovascular and diabetic patients are sometimes referred to the private primary healthcare facilities due to lack of infrastructure in the public health sector |
| Polanczyk & Ribeiro, 2009 | Coronary artery disease in Brazil: contemporary management and future perspectives | Literature review | Cardiovascular diseases are the leading cause of death in Brazil (upper-middle income), mostly due to stroke. | The first specialty unit, the chest pain unit was established by the private sector  Elective procedures were done mainly in the private sector  Due to the lack of government-funded laboratories, patients were referred to private laboratories |
| Subramanian, et al., 2018 | Cost and affordability of non-communicable disease screening, diagnosis and treatment in Kenya: Patient payments in the private and public sectors | Cross-sectional study | NCDs are increasing in Kenya (lower-middle income) | About half of all health facilities in Kenya is managed by private sector.  Care received in the private sector is more expensive than in the public sector |
| Mukherjee, et al., 2011 | Social class related inequalities in household health expenditure and economic burden: Evidence from Kerala, south India | Quantitative | Increased dependence on the private sector in India (lower-middle income) has impoverished and marginalized the poor | Private healthcare facilities are located 10km away from a village in India while government hospitals are located 30 km away. Due to travel challenges, people may choose to utilize private sector |
| Bintabara & Mpondo, 2020 | Preparedness of lower-level health facilities and the associated factors for the outpatient primary care of hypertension: Evidence from Tanzanian national survey | Facility-based cross-sectional study | The Tanzanian (lower-middle income country) government is initiating and adjusting the National NCD prevention and control strategy to ensure access to the affordability and availability of NCD services | The availability of basic diagnostic equipment and medicines for these diseases was significantly lower in public lower-level facilities than in their private counterparts, meaning, the private sector was more ready  Readiness meant staff training and guidelines, basic diagnostic equipment, and basic medications are available |
| Cataife, 2012 | Public versus private treatment of chronic diseases in seniors: Argentina, Brazil, Chile and Uruguay | Literature review | There is disparity of health resources in Latin America but little is known about whether it affects the quality of care provided | Private sector is modern and well endowed while the public sector struggles with financial stress and human and technological resources  In Argentina (upper-middle income), the public sector is less likely to provide treatment for chronic diseases when compared to the private sector or a social security system. Therefore, private healthcare is a necessity in Argentina |
| Pandian, et al., 2007 | Poverty and stroke in India: A time to act | Literature review | Treatment options are limited and unaffordable in developing countries such as India (lower-middle income). Well-organized stroke services are absent, and social determinants of health are barriers to accessing care | Most stroke centers are in the private sector but mostly in the urban areas  Private sector had a coordinated services for stroke and were more efficient than the public sector |
| Wearne, et al., 2019 | Nephrology in South Africa: Not Yet ubuntu | Literature review | The limited workforce in proportion to the need of chronic disease management has led to covert chronic dialysis rationing in the public sector | The private sector has more transplant centers than the public sector  These centers are mostly located in the urban areas impeding access by wide population  Dialysis in the private sector is one of the world’s best serving only 16% of the total population while the remaining 84% of the nephrology patients are concentrated in the public sector |
| Abraham, et al., 2009 | How to make peritoneal dialysis affordable in developing countries | Literature review | Peritoneal dialysis is underutilized in developing countries and the population is severely underserved. | Dialysis is rationed in South Africa due to demands  Innovative plans by the private sector have improved peritoneal dialysis outcomes |

*Innovation Systematic Review*

| **Author/Organization** | **Title:** | **Study Type** | **Context** | **Key Findings** |
| --- | --- | --- | --- | --- |
| Doshi et al., 2021 | Imagining the Future of Primary Care: A Global Look at Innovative Health Care Delivery | Commentary | NCD rates have increased significantly in Mexico (upper-middle income). Even with universal health coverage, there has been limited success partly due to focus on treatment vs. prevention | CASALUD has created a suite of digital tools to help both primary healthcare professionals and patients to properly manage NCDs at a fraction of the cost. |
| Lambert & Kolbe-Alexander, 2013 | Innovative strategies targeting obesity and non-communicable diseases in South Africa: What can we learn from the private healthcare sector? | Review | Overweight and obesity rates are increasing in South Africa (upper-middle income), leading to increased health expenditure from obesity-related illnesses. Most is paid for by private insurance companies | Discovery Health Private insurance incentive-based program shows significant relationship between engagement in wellness activities with lower healthcare expenditure and increase in overall ratio of health foods to total purchases  Program innovated incentive-based health promotion, including visits to dieticians, enrolling in gym programs, purchasing health foods |
| Rockers et al., 2019 | Effect of Novartis Access on availability and price of non-communicable disease medicines in Kenya: a cluster-randomised controlled trial | Cluster-randomized controlled trial | LMICs face NCD burdens, however, management of NCDs are limited due to resources available, including essential medications in Kenya (lower-middle income) | Novartis, a pharmaceutical company applied an innovative business model to provide medications at a significantly reduced price  Novartis access is a program that offers LMICs NCD medications at a wholesale of $1 per treatment per month |
| da Cruz Paula et al., 2020 | Healthcare telemonitoring and business dynamics: challenges and opportunities for SUS | Quali-quantitative approach with exploratory research | Telemonitoring has been documented to be effective for NCD management. Brazilian (upper-middle income) Healthcare system activity in this area has been scarce | Several for-private companies have created business opportunities in the telemonitoring market, giving them monopoly in this area  Several technologies and services are being created using telemonitoring. |
| Saldarriaga et al., 2017 | Point-of-Care Testing for Anemia, Diabetes, and Hypertension: A Pharmacy-Based Model in Lima, Peru | Developed multi-phase training curriculum and evaluated point-of-care testing before and after intervention | In Peru (upper-middle income), NCD burden is sky-rocketing. Part of the innovative delivery model includes provision of care through pharmacies and drugstores (i.e., point of care testing for anemia, hypertension, diabetes) | Private pharmacies provided their clients, infrastructure, and expertise to measure the feasibility of this innovative healthcare delivery to further research in the areas of chronic diseases. |
| Shannon et al., 2019 | Innovating to increase access to diabetes care in Kenya: an evaluation of Novo Nordisk’s base of the pyramid project | Interviews, questionnaires, focus group discussions, site visits, and document reviews from various stakeholders | In Kenya (lower-middle income), diabetes prevalence is increasing and many people struggle to receive basic diabetes care | Novo Nordisk launched a program aiming to strengthen the continuum of diabetes care: increased awareness of diabetes; early diagnosis of diabetes; access to quality care by trained professionals; stable and affordable insulin supply; and improved self-management through patient education  They used an innovative model using the bottom of pyramid (BoP) to provide diabetes care especially insulin only for $5 |
| Tzeel, 2011 | Diabetes Benefit Management: Evolving Strategies for Payers | Review | The burden of type 2 diabetes is unsustainable in LMICs and HICs. 1 in every $10 spent on health care goes towards diabetes management. Appropriate management can mitigate mortality and morbidity rates | Value-based Insurance Design (VBID) is a concept in health insurance where the benefits of care exceed the cost  Certain private health insurance companies, pharmacies benefit managers, and employers have started to adopt this |
| van de Vijver et al., 2013 | Introducing a model of cardiovascular prevention in Nairobi’s slums by integrating a public health and private-sector approach: the SCALE-UP study | Theoretic model design based on cost feasibility analyses | CVDs are leading cause of mortality in Sub-Saharan Africa. This is especially the case for people living in slums within Nairobi, Kenya (lower-middle income) where access to formal healthcare is limited | Cost- effective service delivery package for CVD prevention package was developed by Boston Consulting Group in collaboration with two NGOs for slum areas in Nairobi  Package has 4 components which includes vouchers for free treatment and support group through which participants can acquire subsidize medication  Providing incentives for CHWs, and sending text messages (SMS) to remind patients of clinic appointments, medication use, and healthy lifestyles |
| Hancock et al., 2011 | The private sector, international development and NCDs | Review | NCDs are a global epidemic affecting HICs and LMICs including South Africa (upper-middle income) and Zambia (lower-middle income). This is underserved area which private sector has opportunity to take lead in and become a part of the solution. | A mobile phone platform was developed in South Africa that allows local community caregivers to send patients’ data to doctors for review at the clinic.  PepsiCo made changes to some of their process in the areas of the product (composition, provide more food and beverage choices), marketing (promote healthy lifestyle and make informed decisions), and community (collaborate with both local and global partners to address nutritional challenges).  Rural areas are often beyond reach for healthcare services due to distribution channels, yet commercial products like soft drinks widely available in these areas in countries such as Zambia. *Aidpods*, containers that fits into the crate of bottled drinks, have been created to carry essential medications for NCD management, such as the *polypill.* Distributors are incentivized through small payments at each stage to ensure sustainability.  *Polypill* is a single daily drug that contains several generic drugs to prevent NCDs and can be manufactured for $1 per dose. |
| Medtronic Foundation, n.d. | Addressing Noncommunicable Diseases by Building Capacity, Community, and Commitment | Report | HealthRise Initiative focuses on piloting innovative interventions to improve hypertension and diabetes care and outcome. However, many struggle with sustaining these innovations post-pilot phase. This time, pilot interventions include innovative ways to ensure sustainability | HealthRise Brazil was built into the public health system in collaboration with the Secretary of Health. This implementation method allowed it to stary even without extra funding.  Health Rise India created and tested an electronic health card to gather information on individual NCD health risk during home visits.  They also added a follow-up care module. The government of India adopted it and committed to maintain this application. |
| IFC, 2011 | Public-Private Partnership Stories. India: Andhra Pradesh Radiology | Report | In Andhra Pradesh, India (lower-middle income), access to diagnostic testing like MRI and CT are limited as in the rest of the country. Only a few public hospitals offer such specialized and advanced diagnostic services. | Diagnostics testing for the cost of nearly 50% below market value, that is INR 1700. This enabled the government to provide services to more underserved people within the allocated budget. |
| U.S Department of State | Pink Ribbon Red Ribbon Overview | Factsheet | Breast and cervical cancer are the leading cause of cancer-related death in women in Latin America and Sub-Saharan Africa. | An innovative partnership resulted in steeply discounted cervical diagnostic testing that allows screening of a wide range of people, including people at the bottom of the pyramid (BoP). The partnership also resulted in a customized HPV DNA and HPV DNA test designed for low resource setting. |
| AstraZeneca, 2021 | Healthy Heart Africa | Webpage report on AstraZeneca website | In 2014, Astra Zeneca launched the Health Heart Africa Project to reach the 10 million hypertensive patients in Africa. This was following WHO’s goal to reduce premature cardiovascular death by 25% by 2025. | Astra Zeneca provides branded name anti-hypertensive medications at a significant discounted price. They utilize an innovative business model that is no profit/no loss in order to provide these services. |
| Grancelli, 2005 | Randomised trial of telephone intervention in chronic heart failure: DIAL trial | Randomized controlled trial | Despite advanced medical interventions for heart failure, its burden continues to grow in LMICs like Argentina. Quality of life for those affected is characterized by frequent hospital readmissions due to medication non-adherence, lack of social support, and failure to seek early treatment with worsening symptoms. | This research study was funded by Roche, Boehringer Ingelheim, Pharmacia, Novartis, and Merck Sharp and Dohme in Argentina. The first multicenter trial that achieve same results as the previous ones by applying a simple programme in a large and non-selected population of outpatients with heart failure, in very different clinical settings. |
| Irl et al., 2019 | Culinary Medicine: Advancing a Framework for Healthier Eating to Improve Chronic Disease Management and Prevention | Commentary | Unsustainable increases in NCDs and associated costs necessitated interventions that have high impact and low-cost of for chronic disease management. The new field of culinary medicine offers this | Culinary medicine is an emerging field that combines science of medicine and food preparation and presentation. |

*Knowledge Educator Systematic Review*

| **Author/Organization** | **Title:** | **Study Design** | **Context** | **Key Findings** |
| --- | --- | --- | --- | --- |
| Malekzadeh et al., 2020 | Strengthening research capacity in LMICs to address the global NCD burden | Narrative Report | NCDs are significantly affecting LMICs. To respond, local research becomes important however, research capacity in LMICs is inadequate. | The National Heart, Lung, and Blood Institute (NHLBI) and the United health Group (UHG) collaborated and supported 11 LMICs research centers.  The goal of this initiative was to decrease cardiovascular and respiratory diseases by facilitating a global biomedical researcher network that will do collaborative research, train researchers, and advise on policy. |
| Laar et al., 2019 | Health system challenges to hypertension and related non-communicable diseases prevention and treatment: Perspectives from Ghanaian stakeholders | Qualitative study | Hypertension is prevalent in Ghana (lower-middle income). About 1/3 of people with hypertension were not aware of their diagnosis. Although, hypertension is recognized as emerging, resources are still being allocated to infectious diseases. Current hypertension management is not up to standard | Community-based hypertension Improvement Program (ComHIP) is an initiative launched by Novartis Foundation in collaboration with Ghana Health Service. The program includes evidence-based hypertension management interventions that has been adopted, adapted, and implemented in Ghana. |
| Pastakia et al., 2020 | Framework and case study for establishing impactful global health programs through academia - biopharmaceutical industry partnerships | Literature review with case studies | The healthcare workforce is experiencing shortages. Existing healthcare workers must be trained in other areas as appropriate to attend to more complex health issues | Celgene invested in pharmacy education to help them advance in their skills. Pharmacy technicians were not allowed to dispense drugs while the pharmacist were utilized in outpatient and inpatient settings. Other careers paths were also created in pharmacy that complimented the new additions to the healthcare system. |
| Abdool-Gaffar et al., 2011 | Guideline for the management of chronic obstructive pulmonary disease--2011 update | Review | South Africa (upper-middle income) had highest COPD prevalence, potentially due to higher incidence of smoking, occupational dust exposure, indoor pollution | Private practitioners, together with other pulmonologists from universities, in South Africa, called the COPD Working Group revised the guideline for COPD management based on emerging findings from research that has informed recent recommendations. |
| Patel et al., 2010 | The association between medical costs and participation in the vitality health promotion program among 948,974 members of a South African health insurance company | Evaluation of program | The cost of NCD healthcare services is increasing and private insurance tends to pay majority of the cost. Private Health Insurance companies like *Discovery* in South Africa (upper-middle income) is promoting health within their customers | The Vitality Program, spearheaded by Discovery Private Health Insurance in South Africa has included in their services activities that promote health  Health activities are categorised into 4: fitness-related activities; assessment and screening; healthy choices and health knowledge. |
| Novo Nordisk, 2017 | Base of the Pyramid programme | Report | Diabetes poses a threat to the Chinese community (upper-middle income). The number of people diagnosed with type diabetes in 2010 is expected to double by 2025. | *Novo Nordisk China*, has educational programs for patients living with diabetes. The educational program consists of both promoting awareness of diabetes, management, and prevention. They improve the understanding of how medications can be taken optimally. They launched the *Novocare Hotline*, where patents can call and talk with one of their diabetes management specialists. The main purpose of their NovoCare bus is to promote awareness of diabetes and also provide feedback to those already diagnosed and facilitate educational initiatives as follow-up. The bus features multimedia exhibits where people can learn more about diabetes; patient videos and quizzes, waist measurements, and on-site blood glucose testing. |
| Merck KGaA, 2021 | Merck Foundation Capacity Advancement & Nationwide Diabetes Blue Point Program | Report | There has been a rapid rise NCD-related disability and premature death, with Africa being hit the hardest. But some healthcare systems are unable to address the needs rising | Mercks Capacity Advancement Program (CAP) is a collaborative program with ministries of health, universities, health facilities, local communities across Sub-Saharan Africa.  Merck& Co., Inc provide funding and educational materials for the training of healthcare professionals, medical students education and awareness. Their goal is to expand professional capacity in the areas of research and development, medical education, and community awareness of NCDs. |
| Astra Zeneca, n.d. | Healthy Heart Africa | Post on Companies website | Healthy Heart Africa is a program targeting hypertension. Astra Zeneca has committed to reducing hypertension across Africa. | Astra Zeneca launched a program called *Healthy Heart* Africa and has collaborated with local government and NGOs. As part of their initiative integrate hypertension screening into the continuum of care, Astra Zeneca tested models of care to understand what works for the system and use that to inform future scaling across the region. |
| AVON Global Scholars, n.d. | AVON Global Scholars Program | Post on Avon’s website | Women in LMICs experience greater mortality from breast cancers than their counterparts in HICs. | Scholarships are awarded to outstanding clinicians specialized in breast cancer from around the world to one of Avon’s funded breast cancer centers in the United States. In this program they receive training and preceptorship. |
| Reeves, 2011 | The Private Sector’s Role in Combating NCDs | Blog post on CSIS | Private companies can play a role in managing NCDs globally by strengthening healthcare system capacity to prevent, manage, and control NCDs. They can provide supplemental support through education to compliment the efforts of the public health system | *Medtronic* in collaboration with the Chinese Ministry of Health has opened the Patient Care center in China (upper-middle income country). Not only can patient go to this center for treatment options involving technologies, this center also provides physicians with product and therapy training. Likewise, Medtronic has trained many other physicians in LMICs where there is a shortage of doctors and have increased life saving treatments like smart implants to manage NCDs. |
| Ahmed et al., 2015 | The prevention and management of chronic disease in primary care: Recommendations from a knowledge translation meeting | Narrative study | To effectively manage NCDs, chronic disease management should be integrated into primary healthcare | Recommendations from a knowledge translational meeting will help the sustainability of chronic disease management programs and could be used as a guide for other who want to initiate similar programs. Chronic disease management programs and knowledge translation meeting was financially sponsored by Pfizer. |
| Pfizer Upjohn, 2020 | Leading the Conversation on Noncommunicable Diseases Worldwide: an Evidence-Based Review of Key Research and Strategies to Develop Sustainable Solutions | Webpage on Pfizer website | Impacting factors in NCD progress differs between HICs and LMICs, therefore, it is important to understand target population and develop interventions accordingly | Upjohn, developed an evidence-based framework that is market-specific and accounting for local NCD dynamics. The purpose of this framework is to assist stakeholders in determining appropriate interventions in specific locations. |
| AB InBev, 2018 |  | Progress report | Harmful use of alcohol is a significant factor in developing an NCD(s). | AB InBev, a beer company, has created a global program called the Global Smart Drinking Goals whose aim is to reduce harmful use of alcohol globally by identifying effective, evidence-based programs and policies for public-private partnerships to advance positive social and behavior change. The Foundation works with AB InBev and supports academic and expert partners and researchers, as well as the communities in which the Global Smart Drinking Goals are being implemented. The Foundation currently funds program evaluations to assess the impact of the Global Smart Drinking Goals, and related initiatives, on reducing harmful use of alcohol. |
| Ratzan et al., 2013 | The digital health scorecard: A new health literacy metric for NCD prevention and care | Narrative study | Some causes of NCDs are modifiable and should not lead to premature death, however, there is no globally available, cost-effective, easy to use outcome metric that will push change on all levels yet like it does for other specialties | Johnson and Johnson funded the digital health board and research associated with it. The aim of the scoreboard is to increase public, professional, and policy maker NCD health literacy (the motivation, and ability to access, understand, communicate, and use information to improve health and reduce the incidence of NCD). This tool also provides healthcare stakeholders a simple metric to track progress in reaching NCD target goals. |

*Investment and Finance Systematic Review*

| **Author/Organization:** | **Title:** | **Study design:** | **Context:** | **Key findings:** |
| --- | --- | --- | --- | --- |
| Allen, 2017 | Financing national non-communicable disease responses | Debate | LMIC progress towards SDGs with respect to NCD funding | Novel financing initiatives, commercial entities with objectives that align with NCD agenda, and PPPs can raise additional funds to fight NCDs |
| Anson et al., 2012 | Availability, prices and affordability of the World Health Organization’s essential medicines for children in Guatemala | Cross-sectional analysis using surveys based on WHO/HAI methodology | Guatemala (upper-middle income) obtaining baseline measurements regarding WHO Essential Medicines List for Children | MPRs substantially higher in private sector vs. PROAM; final patient prices approximately 6.41 times higher in private sector than PROAM  Salbutamol MPR was 4.94 in private vs. 1.20 in PROAM |
| Armstrong-Hough et al., 2020 | Variation in the availability and cost of essential medicines for non-communicable diseases in Uganda: A descriptive time series analysis | Prospective, descriptive analysis using surveys | Uganda (low-income) health care is available in private for-profit, private non-profit, and public sectors; essential medicine availability and cost poor in LMICs | >50% of medicines experienced major price fluctuations, with greatest fluctuations occurring in private for-profit sector (UGX 37-17,050). Could be due to dynamic pricing models that ensure profit |
| Ashigbie et al., 2020 | Availability and prices of medicines for non-communicable diseases at health facilities and retail drug outlets in Kenya: A cross-sectional survey in eight counties | Cross-sectional analysis using surveys | Kenya (lower-middle income) launched National Health Policy but have high NCD burden | MPR significantly higher for NCD meds than acute in for-profit drug outlets, greater within provider price variations in private drug outlets vs. public  MPR often higher for NCD meds in private non-profit than private for-profit, but both higher than public sector |
| Beran et al., 2019 | Access to insulin: Applying the concept of security of supply to medicines | Narrative report | LMIC security of supply of generic NCD medicines lower than for communicable diseases | Global insulin market dominated by 3 multinational companies controlling 99% of insulin market value  Manufacturers replace human insulin with more expensive analogues without evidence they’re more effective  Average insulin cost for individual in Zambia 2003 was already US$ 26 in public and US$ 218.40 in private sector |
| Bloom et al., 2011 | The Global Economic Burden of Non-communicable Diseases | Report combining findings of 2 other reports | 42 LMICs that account for 90% of NCD burden in developing regions of world | Cost of “best-buy” interventions for CVD scaled-up response for 2011-2025 is 120 billion USD with projected economic benefit 377 billion USD  Private sector can leverage core business skills, networks and funds to access target populations and offer more innovative products/solutions such as “best-buys”  Collaboration with other sectors allows to capitalize on individual strengths and realize benefits beyond reach of any single entity to ensure necessary resources to manage NCD burden |
| Cameron et al., 2009 | Medicine prices, availability, and affordability in 36 developing and middle-income countries: a secondary analysis | Secondary cross-sectional analysis of using surveys | 36 developing and middle-income countries where medicines account for large portion of health spending and OOP expenditures, especially in private systems | Public sector patient prices generally lower than those in private sector, especially for originator brands  Private sector originator MPR for salbutamol 200-dose inhaler varied between 5.58 in Europe WHO region and 14.26 in Africa WHO region  Private sector lowest-price generics MPR for salbutamol varied between 3.28 in eastern Mediterranean WHO region to 7.19 in Africa WHO region  Public sector LPG salbutamol MPR lower than private sector OB and LPG in all regions, except in western Pacific where private sector LPG was lower by 0.32 |
| Cattaneo & Piemonte, 2021 | Transition Finance Compendium: Challenges and recommendations for the Development Assistance Committee | Report | Developing nations can use Official Development Assistance (ODA) as source of financing, but need exit plan to ensure transition to other forms of financing like private and domestic without gaps | LMIC health systems often lack adequate funding, not designed to manage chronic disease so financial burden falls on patients  Private insurance often underdeveloped in LMICs but role can be critical in financing specialized treatment for high-income segments of population  Frees up tax-based and government-supported scheme resources of low-income households  Planning for transition out of ODA means exploiting potential in early stages, set up market early, engage private sector |
| Dugee et al., 2018149 | Who is bearing the financial burden of non-communicable diseases in Mongolia? | Secondary analysis of multiple data sources including administrative data, WHO STEPS cross-sectional survey, household surveys | Mongolia (lower-middle income) budgetary constraints prevent UHC, growing private sector emerging to meet demands, have core package of essential services funded by state budget and complimentary package financed by social health insurance, OOP payments, donors | Private OOP payments funded almost 2/3 of estimated NCD spending in Mongolia  Private OOP expenditures on major NCDs dominated by richer groups, with OOP expenditures for outpatient services of richest groups 2.4x that of poorest |
| Dutta & Ly, 2018 | Financing the health systems of the future: A proposed framework for including non-communicable diseases | World Bank blog post | LMIC health sector reform gaining traction but need to finance health systems that address pressing needs such as NCDs; diverse funding modalities can help move conversation forward for NCD financing when governments perceive issue as too costly | Private health insurance doesn’t exist in many low-income settings, but there is need and market for it within higher income groups  Private insurance can fill financing gap by providing critical care for higher income segments, allowing rational targeting for tax-based and government-supported scheme resources for lower income segments  Innovative PPPs critical to expand access and offer solutions to various aspects of NCD financing challenges |
| El-Saharty S, et al., 2013 | Tackling Noncommunicable Diseases in Bangladesh: Now Is the Time | Report | Bangladesh (lower-middle income) is undergoing rapid demographic and epidemiologic transition with shift in disease burden to NCDs, which can have huge impact on financial vulnerability especially for the poor and can indirectly impact economy | Private spending was high with nearly 86% of private expenditure OOP  Align payments for private health insurance programs covering employees of large private companies with provider performance in NCD risk factors and prevention  Outsource expensive and high-tech clinical interventions to private sector may be more cost effective  Private insurance can be costly but can also mitigate high prices of treatment, reduce public expenditure on healthy by taking part of burden with expectation for high quality services |
| Ganju et al., 2020 | Systemic Solutions for Addressing Non-Communicable Diseases in Low- and Middle-Income Countries | Review of deliberations of the Think Tank from Expert Forum on NCDs in Emerging Nations | LMICs have disproportionate number of premature deaths due to LMICs, UNHLM 2014 emphasized need for strengthening health systems prioritizing NCDs, Pfizer launched Upjohn in 2018 as division to fight against NCDs | Cost of healthcare rising more rapidly than economic growth so need “value-based healthcare”  “Best Buy” NCD interventions are low cost but can return billions of dollars to economy  Multisectoral approach with private sector needed to ensure NCD resources evenly distributed and accessible; need public- and private-sector collaboration to achieve targeted outcomes  Interventions targeting training, information sharing, patient empowerment struggle with sustainability, but good opportunity for private sector funding |
| Heidari et al., 2019 | Availability, pricing and affordability of selected medicines for noncommunicable diseases | Cross-sectional analysis using surveys based on WHO/HAI methodology | Iran (upper-middle income) government controls pharmaceutical production and importation to keep costs contained, all pharmacies provide medicines from same distributors, and all Iranians use some form of insurance system to compensate drug expenses | Median price for generic versions 1.19 times reference, overall procurement price for 11 originator brands 4.02 times international reference  Prices for originator brands 3.5 times higher than reference price in private retail pharmacies, patient prices for most generics close to reference price  Originator brand prices 3.99 times higher than reference price in private pharmacies located in public hospitals, and most sold generics 1.19 times the reference price  Prices did not vary considerably between public, private and private within hospitals due to Iran’s successful model to control prices across sectors |
| Holt et al., 2017153 | Winning in Nigeria: Pharma’s next frontier | Narrative report | Nigeria (lower-middle income) experienced strong economic growth which sparked enthusiasm about pharmaceutical market opportunities, but now need different strategies to support disease burden shift towards being NCD-dominated | Private health insurance in Nigeria makes up only 5% of total health expenditures  <5% of households can pay for ethical drugs through OOP or private health insurance  Pharmaceutical companies should design trade terms and affordability strategies to mitigate price increases that occur through transport, distribution, and retail mark-up to help manage health spending |
| Jones, 2021 | Global action on financing cervical cancer elimination: Funding secondary prevention services in low resource settings | Report including review and in-depth interviews | Women in LMIC disproportionately affected by cervical cancer-related morbidity/mortality, and there are challenges in low-resources settings surrounding financial sustainability of efforts to eliminate cervical cancer as public health problem | Development bank lending provides funding through blended financing structure including stakeholders from private sector (e.g., IsDB partnership with International Energy Agency launched “Saving Women’s Lives from Cancer’)  Development impact bonds/outcomes-based financing/social impact bonds use initial upfront investment from private sector which outcome funder (government, development agency) reimburses once desired outcome achieved  PPPs catalyze new services and leverage public and private investments  Innovative finance solutions complement insufficient domestic funding in low-resource settings, especially those engaging private sector  Channel corporate social responsibility spending into priority health areas and prioritize best buy options |
| Kanzler & Ng, 2012 | The Future of Public and Private Health Care Insurance in Asia | Book chapter | In emerging and developing countries in Asia, governments have primary responsibility to provide care and private insurance mainly niche market reserved for those with expendable income, but spiraling healthcare costs due to increasing NCD burdens suggest governments should consider private insurance’s role | Most Asian countries currently mandate state coverage but allow patients to purchase private insurance as supplement  Private insurance can reduce OOP expenses, improve quality or expand depth of services provided/reimbursed for, increase access to healthcare for large segments of populations lacking formal coverage  Ability of private insurers to manage costs limited due to competition to provide better services so won’t automatically control rising costs but can produce financial benefits for public sector  Free latent demand for higher level of care by assuming reimbursement responsibility for more expensive treatments, relieving financial pressure on cash-strapped governments |
| Khuluza & Haefele-Abah, 2019138 | The availability, prices and affordability of essential medicines in Malawi: A cross-sectional study | Cross-sectional analysis using surveys based on WHO/HAI methodology | Malawi (low-income) healthcare covered by public and private sector, but public sector has cost-covering interventions which are being challenged by rapid population growth | Overall MPR was 1.11 for wholesalers, 2.54 in CHAM, 2.70 in retail pharmacies, 4.01 in private sector  Diazepam MPR 5g/mL injection varied considerably: 1.63 wholesale, 5.39 retail pharmacies, 6.17 CHAM, 12.01 private clinics  Insulin MPR 100 IU/mL soluble injection, 10 mL: 1.66 wholesale, 3.49 retail pharmacies, 1.81 CHAM, 2.21 private clinic  Methyldopa MPR 250 mg tab: 1.10 wholesale, 2.23 retail pharmacies, 1.70 CHAM, 1.77 private clinic |
| Kishore et al., 2015 | Overcoming obstacles to enable access to medicines for noncommunicable diseases in poor countries | Narrative report | LMICs have less access to NCD medicines due to high OOP expenditures and financial burdens | High prices for patented cancer drugs like Sprycel and Herceptin, but entry of generic would lower prices significantly  Sorefenib had generic version brought to market in India when Controller General of Patents, Designs and Trademarks issued compulsory license so that parties other than Bayer could produce and sell at more affordable price  Private sector markups ranged from 2% to 380% across LMICs  Current R&D from pharmaceutical companies puts many essential medicines out of reach for poorer populations  Motivations of pharmaceutical industries to develop NCD meds focus on profits, campaign to undermine legislation to allow generics. Suppliers can negotiate voluntary licenses. |
| Ladusingh L et al., 2018139 | Triple burden of disease and out of pocket healthcare expenditure of women in India | Cross-sectional analysis using nationally representative survey (NSSO) | India (lower-middle income) faces high NCD burden and OOP expenditures, especially in women who face triple burden of disease | Economic burden for treatment of NCDs is highest  Seeking healthcare in private health facilities much more expensive than public facilities with OOP expenditures for NCDs being 2.3x higher than public  Private hospital inpatient mean medical expenditure highest for NCDs (Rs 4928 in private vs. Rs 2580 in public) |
| Lambert & Kolbe-Alexander, 2013103 | Innovative strategies targeting obesity and non-communicable diseases in South Africa: What can we learn from the private healthcare sector? | Review | South Africa (upper-middle income) has >50% adult women and 30% adult men overweight or obese thus country’s largest private health insurer, Discover Health, launched incentive-based Vitality programme | Programme subsidized gym memberships (up to 80%), provided cashback on healthy foods  Members accumulate points through increased participation in programme to claim substantial discounts on various purchases and services  2009 Vitality South Africa HealthyFood benefit offered cash rebate of up to 25% on >10,000 health food items in major national retail supermarket chain  Significant inverse relationship between level of engagement in Vitality program and medical claims |
| Lekshmi et al., 2014143 | Study on Availability and Affordability of Anti Hypertensive Medicines in the State of Kerala | Prospective, observational study using surveys based on WHO/HAI methodology | Kerala, India (lower-middle income) has high NCD morbidity, leaving public sector incapable of meeting demands thus requiring private sector use | All hypertension treatments were available and affordable at KCP pharmacies, and more expensive in private sector  Maximum mean retail prices of 30-day treatments for all stages of hypertension were higher in private sector than public KCP pharmacies |
| Mendis & Chestnov, 2013 | Policy reform to realize the commitments of the Political Declaration on noncommunicable diseases | Report based on findings from United Nations High-Level Meeting on NCDs 2011 | LMICs face challenge of NCDs in pursuit of socioeconomic development, choice of NCD interventions for scaling up action depends on feasibility and affordability of implementation in these settings | Tax increases on unhealthy commodities reduce consumption and fund NCD healthcare  Political Declaration suggests engaging all stakeholders, even private sector, to improve affordability, accessibility and maintenance of diagnostic equipment and technology, medicines, mobilizing adequate, sustainable and predictable financial resources  Private sector can provide financial and technical support and training  Invest in “best buy” interventions for which cost is low but economic benefits high |
| Mendis S, et al., 200146 | The availability and affordability of selected essential medicines for chronic diseases in six low- and middle-income countries | Cross-sectional analysis using surveys based on WHO/HAI methodology | LMICs carry global burden of disease form NCDs, with 80% of deaths, with many reasons for why medicines are not used more often | Cost of innovator products in Malawi and Sri Lanka private sector 3x more than generics  Minimal variation in prices among private outlets in Nepal and Pakistan  Prices in private outlets varied considerably in Malawi  Private sector prices 66.3% higher than public sector in Nepal  Total add-on costs applied to manufacturer price ranged from 18% in Pakistan to >90% in Malawi  Throughout distribution chain, price increases from wholesale and retail mark-ups  Monitoring of supply chain and regulating mark-ups may help lower prices  Pakistan regulates price through enforced maximum mark-ups and selling prices |
| Mhlanga & Suleman, 2014 | Price, availability and affordability of medicines | Cross-sectional analysis using surveys inspired by WHO/HAI methodology | Swaziland/Eswatini (lower-middle income) has 63% of population living below upper poverty line and government, private sector, NGOs and faith-based organizations are involved in providing health services. Medicines free at point of delivery in public sector | Originator brand products in private sector were priced 32.4x international reference prices  In private sector, originator brands cost 473% more, on average, than generic equivalents  Median MPR for originator brand products in private sector was 41.06, for LPGs it was 8.67  Retail mark-up contribution to final price was highest mark-up observed ranging from 31%-53%  Metformin had largest retail mark-up contribution to final price with wholesale mark-up between 20%-29%  Metformin OB had highest cumulative mark-up at 440.27% |
| Moodie et al., 2013 | Profits and pandemics: Prevention of harmful effects of tobacco, alcohol, and ultra-processed food and drink industries | Narrative report | LMICs are seeing rise in sales of unhealthy commodities and these industries are major drivers of NCD epidemics globally, but UN High-Level Meeting on NCDs called for multisectoral action including private sector and potentially unhealthy commodity industries | Funding from transnational food and beverage corporations biases research, as more likely to have conclusion favourable to financial interests of sponsoring company  Suggest unhealthy commodity industries should have no role in formation of national or international NCD policy  Despite common reliance on industry self-regulation and PPPs, there’s no evidence of their effectiveness or safety  Should be wary of funding from unhealthy commodity industries as COIs and ulterior motives prevalent |
| Prescott & Stibbe, 2017 | Better Together: Unleashing the Power of the Private Sector to Tackle Non-Communicable Diseases | Report | Globally, SDGs called for action across all sectors, and SDG to reduce NCDs specifically requires all-of-society approach due to scale of epidemic, including collaboration with the private sector | Novo Nordisk established R&D strategy which identifies diabetes and obesity as areas to invest in for drug innovation  Companies can sponsor gym subscriptions or bicycle purchases for employees  Private sector invest in R&D for innovation in NCD treatment, including new technology  Private sector invest in workplace wellness programs, health cost curtailment  Essential to understand motivation or business case motivating companies beyond improving health |
| Rahman et al., 2013 | Health-Related Financial Catastrophe, Inequality and Chronic Illness in Bangladesh | Cross-sectional analysis using 3-stage cluster sampled household surveys | Bangladesh (lower-middle income) has high proportion households incurring catastrophic health expenditures and limited risk sharing mechanisms, and is facing double burden of disease due to NCDs | Risk of financial catastrophe and level of OOP payments higher for users of inpatient, outpatient public and private facilities, respectively, compared to using self-medication or traditional healers  Health insurance can protect families against health shocks that increase healthcare needs and economic shocks that reduce capacity to finance healthcare |
| Sado & Sufa, 2016 | Availability and affordability of essential medicines for children in the Western part of Ethiopia: implication for access | Cross-sectional analysis using surveys based on WHO/HAI methodology | Ethiopia (low-income) lacks data on availability and affordability of essential medicines for children. Access has been a big challenge previously in developing countries | Patient prices were 36% times higher in private sector than public sector  MPR for salbutamol 100 mcg/dose inhaler 0.80 in public sector, 1.54 in private sector  Notable variability in prices across drug outlets in private sector potentially due to high market competition |
| Schmutz et al., 2019166 | Mapping the global cancer research funding landscape | Review | Global context, as NCDs are on rise globally and are major challenge for global economy, with cancer specifically drawing interest from wide spectrum of research funders | Capacity of country to provide funding for research closely linked to its economic prowess and educational status  Total number of cancer research funding sources has more than doubled since 2008, number of private for-profit companies has quadrupled, accounting for 17% of entities funding cancer research  Speculate funding from private for-profit entities underrepresented in publications |
| Singh et al., 2016 | Assessment of Universal Healthcare Coverage in a District of North India: A Rapid Cross-Sectional Survey Using Tablet Computers | Cross-sectional analysis using rapid tablet computer-based surveys | India (lower-middle income) pre-existing national surveys are large but expensive, logistically difficult and time-consuming this difficult to track progress towards health goals when data not regularly available | Mean expenditure on chronic illnesses was 2328 USD in private sector, and 1040 USD in government sector  Expenditure in government health facilities was less than in private health facilities for all events included in study which required healthcare |
| Subramaniam et al., 2018 | Cost and affordability of non-communicable disease screening, diagnosis and treatment in Kenya: Patient payments in the private and public sectors | Interviews with NCD specialists, review of public literature and data | Kenya (lower-middle income) has rising NCD prevalence, with CVD being leading cause of NCD mortality, thus government launched 5-year National NCD Strategy in 2015 | Cost for screening procedures for early NCD detection range from US$3.90-US$10.50 in public, US$18-US$36 in private  Patient costs for diabetes, asthma, hypertension, breast and cervical cancer, chronic kidney disease, and stroke and heart conditions higher in private sector than public sector |
| Tripathy & Prasad, 2018 | Cost of diabetic care in India: An inequitable picture | Secondary analysis of NSSO 2014 survey | India (lower-middle income) has very high and growing diabetes public health problem, and low public health expenditures leading to high OOP expenditures, with healthcare financing and delivery largely left to private sector | Median private sector OOP hospitalization expenditure 4x higher than public sector (231 USD vs. 57 USD)  Significantly higher prevalence of catastrophic expenditure found when care sought from private facility vs. public (23% vs. 7%)  Indirect costs nearly 2.5x higher in public sector vs. private  Median outpatient OOP expenditure due to diabetes was 8 USD in private sector vs. 3 USD in public |
| Tusubira et al., 2020 | Accessing medicines for non-communicable diseases: Patients and health care workers’ experiences at public and private health facilities in Uganda. | Qualitative analysis using in-depth key informant interviews and focus group discussions | Uganda (low-income) lacks data about NCD medicine barriers, public hospitals are government-owned and provide free services while private sector is varied and diverse with for-profit financed through direct payment or private insurance; no public insurance model | Financial factors (high costs of medicines, limited insurance coverage) were barriers in private facilities  Health insurance (private only in Uganda) identified as both facilitator and barrier in PFP facilities  Health insurance facilitated access to medicines at PFP facilities among those insured but those with limited/expired coverage faced high costs  Despite barrier of high cost, private facilities sought out due to perceived higher quality services |
| Shellaby & Henshall, 2018 | Confronting the Investment Gap in Non-Communicable Disease Treatment and Care | Report | LMICs face significant financing gap for NCD treatment and care so need to find sustainable solutions to close gap | Blended finance uses non-return and return-seeking capital to attract private capital to socially impactful investments with weak risk-return profiles, public funds and philanthropic grants de-risk investments  Private sector investment has potential to be important source of capital to fill NCD financing gap, given strain on domestic spending  Despite availability of funding, private sector investment accounts for only 5% of annual health infrastructure investment in LMICs  Private investors tend to target private providers of high-cost care unaffordable for most of population |
| UNICEF, 201922 | Programme Guidance for Early Life Prevention of Non-Communicable Diseases | Report | LMICs disproportionately affected by NCDs with >85% of premature deaths thus need to engage multiple sectors to reduce societal, environmental and behavioural NCD risks | WHO Independent Global High-Level Commission on NCDs recommends UN agencies establish multi-donor catalytic Trust Fund to support countries with NCD prevention/control, attracting investments from various sectors in health portfolios (agriculture, food production, innovation to reduce NCD burden)  Partnerships with private sector & PPPS can yield funding to reduce NCD risk |
| FAO et al., 2020 | The State of Food Security and Nutrition in the World 2020. Transforming food systems for affordable healthy diets | Report | LMIC populations may find healthy diets unaffordable as cost exceeds international poverty line, so need to increase affordability as unhealthy diets are leading NCD risk factor | Transport/supply chain costs are major bottleneck in improving affordability of healthy diets, so improvement requires private sector investment  Improve agricultural productivity through private sector investment in agricultural research, technology transfer and tech assistance  Governments can deploy investment decisions via co-investing, taxing, subsidizing or regulating to encourage private sector investment in healthy diets  Private sector investments can increase agricultural productivity, reduce food losses, and enhance efficiencies in food storage, processing, packaging, distribution, marketing |
| van Mourik et al, 2010 | Availability, price and affordability of cardiovascular medicines: A comparison across 36 countries using WHO/HAI data | Secondary cross-sectional analysis of surveys based on WHO/HAI methodology | 36 developing countries, as global CVD burden continues to rise and pharmaceutical use for secondary CVD prevention found to be insufficient in LMICs | For lowest price generics, private sector was on average more expensive for all medicines  On average, affordability was better in private sector for both lowest price generics and originator brands, but when countries were matched private sector was less affordable |
| World Bank, 2013147 | The growing burden of non-communicable diseases in the Eastern Caribbean | Working paper | Organization of Eastern Caribbean States faces growing NCD burden due to epidemiological transitions; NCDs impose large economic burden | Jamaica pharmaceutical companies involved in National Health Fund (NHF) which provides free or subsidized medicines to NCD patients, reducing costs of health service delivery  NCD patients in St. Lucia spent 5x more in private outpatient care health facilities vs. public  Private OOP expenditure for NCDs in St. Lucia 4x higher than public sector |
| WHO, 2013 | Global Action Plan for the Prevention and Control of Noncommunicable Diseases 2013-2020 | Report | LMICs bear 86% of burden of premature NCD deaths, leading to cumulative economic losses of US$ 7 trillion between 2011-2025 | Utilize multi-sectoral financing including private sector sources to strengthen provision of adequate, predictable and sustained resources for NCD prevention/control  International partners can facilitate mobilization of adequate, predictable and sustained financial resources  Improve efficiency of resource utilization through synergy of action, integrated approaches and shared planning across sectors |
| WHO, 2019 | It’s time to walk the talk: WHO independent high-level commission on noncommunicable diseases final report | Report | Many countries lagging in implementing NCD strategies, with limited progress made to secure effective and meaningful commitments from private sector | Recommend establishing MDTF to respond to country demands for international assistance, engage private sector, mobilize multilateral funding, and build technical capacity to fight NCDs |
| The Advisory Group on the Governance of the Private Sector for UHC, 2020 | Engaging the private health service delivery sector through governance in mixed health systems. | Report | Globally, all countries struggle with UHC especially due to increasing NCD prevalence | Health insurance can provide some protection, but not suitable for non-clinical modalities of NCDs especially when entrusted to private actors  International financing (development bonds, private investment, innovative funding streams) can be leveraged to catalyze NCD service delivery  Governments should define private sector role in financing and resource redistribution  Private sector financing increasingly targeted to certain segments of population with social impact agenda |
| You et al., 2019 | Measuring availability, prices and affordability of ischaemic heart disease medicines in Bangi, Selangor, Malaysia | Cross-sectional analysis of surveys based on WHO/HAI methodology | Malaysia (upper-middle income) | Final patient prices for lowest priced generics and originator brands were 10.77 and 24.09 times their international reference prices, respectively. OB cost 63.63% more than generic equivalents, on average  Good Pharmaceutical Trade Practice (GPTP) guideline published to promote standard price and bonus scheme to distribution channels and healthcare providers, but adherence poor as execution by pharmaceutical organizations voluntary |

**Appendix D:** Quality Assessment

*PPP Systematic Review*

| **Author:** | **Abstract & title:** | **Introduction & aims:** | **Method & data:** | **Sampling:** | **Data analysis:** | **Ethics & bias:** | **Results:** | **Transferability or generalizability:** | **Implications & usefulness:** | **Total:** | **Grade:** |
| --- | --- | --- | --- | --- | --- | --- | --- | --- | --- | --- | --- |
| Alizadeh et al., 2020 | 4 | 3 | 3 | 2 | 4 | 3 | 3 | 2 | 4 | 28 | B |
| Silva et al., 2017 | 3 | 3 | 2 | 3 | 2 | 1 | 4 | 3 | 3 | 24 | B |
| Das et al., 2017 | 4 | 4 | 4 | 4 | 4 | 4 | 4 | 4 | 4 | 36 | A |
| Goroff & Reich, 2010 | 4 | 4 | 1 | 1 | 1 | 1 | 4 | 2 | 4 | 22 | C |
| Hawkes & Buse, 2011 | 1 | 4 | 1 | 1 | 1 | 1 | 4 | 2 | 3 | 18 | C |
| HCC, 2017 | 3 | 4 | 3 | 3 | 2 | 1 | 4 | 4 | 4 | 28 | B |
| Hospedales & Jane-Llopis, 2011 | 2 | 4 | 1 | 3 | 1 | 1 | 3 | 3 | 3 | 21 | C |
| Johnson et al., 2018 | 4 | 4 | 2 | 3 | 2 | 1 | 3 | 4 | 4 | 27 | B |
| Jones, 2021 | 4 | 4 | 3 | 3 | 2 | 1 | 4 | 4 | 4 | 29 | B |
| Kraak et al., 2011 | 4 | 4 | 3 | 3 | 2 | 3 | 4 | 3 | 4 | 30 | A |
| Moodie et al., 2013 | 4 | 3 | 2 | 1 | 1 | 1 | 3 | 2 | 3 | 20 | C |
| Moscetti & Taylor, 2015 | 4 | 4 | 3 | 3 | 1 | 1 | 3 | 3 | 4 | 26 | B |
| Oluwole & Kraemer, 2013 | 4 | 4 | 2 | 3 | 1 | 1 | 4 | 3 | 4 | 26 | B |
| Ota et al., 2018 | 4 | 4 | 3 | 3 | 2 | 4 | 4 | 3 | 3 | 30 | A |
| Prescott & Stibbe, 2017 | 1 | 4 | 3 | 1 | 1 | 1 | 4 | 2 | 4 | 21 | C |
| Shannon et al., 2019 | 4 | 4 | 4 | 4 | 4 | 4 | 4 | 4 | 4 | 36 | A |
| Thow et al., 2018 | 4 | 4 | 4 | 3 | 4 | 3 | 3 | 3 | 3 | 31 | A |
| Trager, 2020 | 3 | 2 | 1 | 1 | 1 | 1 | 4 | 2 | 3 | 18 | C |
| UNICEF, 2019 | 2 | 4 | 1 | 1 | 1 | 1 | 4 | 2 | 4 | 20 | C |
| UNICEF, 2020 | 4 | 2 | 1 | 3 | 1 | 1 | 4 | 4 | 4 | 24 | B |
| World Economic Forum, 2013 | 1 | 3 | 1 | 3 | 1 | 1 | 4 | 3 | 3 | 20 | C |
| WHO, 2013 | 1 | 3 | 2 | 3 | 1 | 1 | 4 | 4 | 4 | 23 | C |
| WHO, 2016 | 4 | 4 | 2 | 3 | 1 | 1 | 4 | 3 | 4 | 26 | B |
| WHO GCM/NCD Working group, 2018 | 2 | 3 | 3 | 3 | 1 | 1 | 3 | 3 | 3 | 22 | C |
| WHO, 2019 | 1 | 4 | 2 | 1 | 1 | 1 | 4 | 2 | 4 | 20 | C |

*Governance and Policy Systematic Review*

| **Author:** | **Abstract & title:** | **Introduction & aims:** | **Method & data:** | **Sampling:** | **Data analysis:** | **Ethics & bias:** | **Results:** | **Transferability or generalizability:** | **Implications & usefulness:** | **Total:** | **Grade:** |
| --- | --- | --- | --- | --- | --- | --- | --- | --- | --- | --- | --- |
| Ben Romdhane et al., 2015 | 3 | 4 | 3 | 3 | 3 | 2 | 4 | 3 | 4 | 29 | B |
| Bergman et al., 2012 | 3 | 3 | 1 | 1 | 1 | 1 | 4 | 2 | 4 | 20 | C |
| Bhojani et al., 2014 | 4 | 3 | 3 | 3 | 4 | 4 | 4 | 4 | 4 | 33 | A |
| Buse et al., 2017 | 4 | 4 | 3 | 3 | 3 | 1 | 4 | 4 | 4 | 30 | A |
| Carriedo et al., 2021 | 4 | 4 | 3 | 4 | 4 | 1 | 4 | 4 | 4 | 32 | A |
| Casswell, 2013 | 4 | 2 | 1 | 1 | 1 | 1 | 4 | 2 | 3 | 19 | C |
| Casswell, 2019 | 2 | 2 | 1 | 1 | 1 | 1 | 3 | 2 | 2 | 15 | C |
| Cetthakrikul et al., 2019 | 4 | 4 | 4 | 4 | 4 | 3 | 4 | 4 | 4 | 35 | A |
| Coriakula et al., 2018 | 4 | 4 | 4 | 4 | 4 | 4 | 4 | 4 | 4 | 36 | A |
| George, 2018 | 4 | 4 | 1 | 1 | 1 | 1 | 4 | 2 | 4 | 22 | C |
| Gomez, 2019 | 4 | 4 | 4 | 4 | 4 | 3 | 4 | 4 | 4 | 35 | A |
| Gortmaker et al., 2012 | 4 | 3 | 1 | 1 | 1 | 1 | 4 | 2 | 4 | 21 | C |
| Jaichuen et al., 2018 | 4 | 4 | 4 | 4 | 4 | 4 | 4 | 4 | 4 | 36 | A |
| Khayatzadeh-Mahani et al., 2018 | 1 | 2 | 1 | 1 | 1 | 1 | 3 | 2 | 3 | 15 | C |
| Lauber et al., 2020 | 4 | 4 | 4 | 4 | 4 | 2 | 4 | 4 | 4 | 34 | A |
| Mariath & Martins, 2020 | 2 | 3 | 2 | 2 | 2 | 1 | 3 | 2 | 3 | 20 | C |
| Mialon et al., 2016 | 4 | 4 | 4 | 4 | 4 | 4 | 4 | 4 | 4 | 36 | A |
| Mialon & Gomes, 2019 | 4 | 4 | 3 | 4 | 3 | 1 | 4 | 4 | 4 | 31 | A |
| Mialon et al., 2020 | 4 | 4 | 4 | 4 | 4 | 4 | 4 | 4 | 4 | 36 | A |
| Mialon et al., 2021 | 4 | 3 | 3 | 3 | 3 | 4 | 4 | 4 | 4 | 32 | A |
| Moodie et al., 2013 | 4 | 4 | 1 | 1 | 1 | 1 | 4 | 2 | 4 | 22 | C |
| Moscetti & Taylor, 2015 | 4 | 4 | 1 | 1 | 1 | 1 | 4 | 2 | 4 | 22 | C |
| Myers et al., 2017 | 4 | 3 | 2 | 3 | 1 | 1 | 3 | 2 | 3 | 22 | C |
| Oladepo et al., 2018 | 4 | 4 | 4 | 4 | 4 | 4 | 4 | 4 | 4 | 36 | A |
| Sacks et al., 2015 | 4 | 3 | 4 | 4 | 4 | 1 | 3 | 4 | 3 | 30 | A |
| Sanni et al., 2018 | 4 | 4 | 4 | 3 | 4 | 4 | 4 | 3 | 4 | 34 | A |
| Suzuki et al., 2021 | 4 | 4 | 4 | 4 | 4 | 4 | 4 | 4 | 4 | 36 | A |
| Tangcharoensathien et al., 2019 | 4 | 4 | 1 | 1 | 1 | 1 | 4 | 2 | 4 | 22 | C |
| Thow et al., 2018 | 4 | 4 | 4 | 4 | 4 | 3 | 3 | 4 | 4 | 34 | A |
| Williams, 2015 | 4 | 3 | 3 | 3 | 3 | 1 | 3 | 3 | 4 | 27 | B |
| WHO, 2016 | 4 | 4 | 2 | 3 | 1 | 1 | 4 | 3 | 4 | 26 | B |
| WHO, 2020 | 4 | 3 | 2 | 3 | 1 | 1 | 4 | 3 | 4 | 25 | B |
| WHO, 2021 | 4 | 4 | 2 | 3 | 1 | 1 | 4 | 3 | 4 | 26 | B |

*Healthcare Provision Systematic Review*

| **Author:** | **Abstract & title:** | **Introduction & aims:** | **Method & data:** | **Sampling:** | **Data analysis:** | **Ethics & bias:** | **Results:** | **Transferability or generalizability:** | **Implications & usefulness:** | **Total:** | **Grade:** |
| --- | --- | --- | --- | --- | --- | --- | --- | --- | --- | --- | --- |
| Abraham, et al., 2009 | 3 | 4 | 1 | 1 | 1 | 1 | 1 | 3 | 4 | 20 | C |
| Agarwal, 2005 | 1 | 4 | 1 | 1 | 2 | 1 | 2 | 4 | 4 | 20 | C |
| Angwenyi et al., 2020 | 4 | 4 | 4 | 4 | 4 | 4 | 4 | 3 | 4 | 32 | A |
| Ashigbie et al., 2020 | 4 | 4 | 4 | 2 | 3 | 3 | 3 | 3 | 3 | 18 | C |
| Atre, 2015 | 3 | 4 | 4 | 2 | 3 | 1 | 4 | 4 | 4 | 30 | A |
| Balasubramaniam et al., 2014 | 4 | 4 | 4 | 2 | 3 | 3 | 4 | 3 | 3 | 20 | C |
| Bhojani et al., 2012 | 1 | 3 | 2 | 1 | 2 | 2 | 3 | 3 | 3 | 30 | A |
| Bigdeli et al., 2016 | 4 | 4 | 4 | 4 | 3 | 4 | 3 | 3 | 3 | 26 | B |
| Bintabara & Mpondo, 2020 | 4 | 4 | 4 | 4 | 4 | 4 | 4 | 3 | 3 | 30 | A |
| Bissell et al., 2016 | 4 | 4 | 2 | 1 | 1 | 1 | 3 | 3 | 3 | 30 | A |
| Cataife, 2012 | 3 | 3 | 2 | 2 | 3 | 1 | 3 | 3 | 4 | 18 | C |
| Dabare et al., 2014 | 4 | 4 | 4 | 3 | 4 | 3 | 4 | 4 | 4 | 23 | C |
| Mukherjee, et al., 2011 | 4 | 4 | 4 | 4 | 4 | 1 | 4 | 3 | 4 | 32 | A |
| Musinguzi et al., 2015 | 3 | 4 | 4 | 4 | 4 | 4 | 4 | 3 | 4 | 27 | B |
| Pandian, et al., 2007 | 4 | 4 | 2 | 2 | 2 | 1 | 4 | 3 | 4 | 27 | B |
| Polanczyk & Ribeiro, 2009 | 3 | 4 | 2 | 2 | 2 | 1 | 3 | 3 | 3 | 27 | B |
| Rawal et al., 2020 | 3 | 4 | 4 | 4 | 4 | 3 | 4 | 4 | 4 | 23 | C |
| Subramanian, et al., 2018 | 4 | 4 | 4 | 3 | 3 | 1 | 3 | 3 | 3 | 26 | B |
| Syed et al., 2018 | 3 | 4 | 3 | 3 | 3 | 1 | 4 | 3 | 4 | 32 | A |
| Tusubira et al., 2020 | 4 | 4 | 4 | 4 | 4 | 4 | 4 | 3 | 4 | 32 | A |
| Wearne, et al., 2019 | 4 | 4 | 1 | 1 | 1 | 1 | 4 | 3 | 4 | 30 | A |
| Wirtz et al., 2018 | 3 | 4 | 4 | 4 | 4 | 1 | 3 | 3 | 4 | 27 | B |

*Innovation Systematic Review*

| **Author:** | **Abstract & title:** | **Introduction & aims:** | **Method & data:** | **Sampling:** | **Data analysis:** | **Ethics & bias:** | **Results:** | **Transferability or generalizability:** | **Implications & usefulness:** | **Total:** | **Grade:** |
| --- | --- | --- | --- | --- | --- | --- | --- | --- | --- | --- | --- |
| AstraZeneca, 2021 | 1 | 2 | 1 | 1 | 1 | 1 | 1 | 2 | 3 | 13 | C |
| Da Cruz Paula, et al., 2020 | 4 | 4 | 4 | 4 | 4 | 1 | 4 | 3 | 4 | 32 | A |
| Doshi, et al., 2021 | 3 | 4 | 1 | 1 | 1 | 1 | 4 | 4 | 4 | 23 | C |
| Grancelli, 2005 | 4 | 4 | 3 | 4 | 3 | 2 | 4 | 3 | 4 | 30 | A |
| Hancock, et al., 2011 | 4 | 3 | 1 | 1 | 1 | 1 | 1 | 4 | 4 | 20 | C |
| IFC, 2011 | 1 | 3 | 1 | 1 | 1 | 1 | 1 | 3 | 3 | 15 | C |
| Irl B., H. *et al, 2019* | 3 | 4 | 3 | 2 | 4 | 3 | 4 | 4 | 4 | 31 | A |
| Lambert & Kolbe-Alexander, 2013 | 4 | 3 | 3 | 1 | 3 | 1 | 4 | 4 | 4 | 27 | B |
| Medtronic Foundation, n.d. | 4 | 4 | 1 | 1 | 1 | 1 | 1 | 4 | 4 | 21 | C |
| Rockers, et al., 2019 | 4 | 4 | 4 | 4 | 4 | 3 | 1 | 1 | 1 | 26 | B |
| Saldarriaga, et al., 2017 | 3 | 4 | 4 | 3 | 4 | 3 | 4 | 3 | 4 | 32 | A |
| Shannon, et al., 2019 | 4 | 4 | 4 | 1 | 4 | 3 | 4 | 3 | 3 | 30 | A |
| Tzeel, 2011 | 4 | 4 | 1 | 1 | 1 | 1 | 1 | 2 | 3 | 18 | C |
| U.S. Department of State, 2011 | 1 | 3 | 1 | 1 | 1 | 1 | 1 | 2 | 3 | 14 | C |
| van de Vijver, et al., 2013 | 4 | 4 | 3 | 2 | 3 | 1 | 3 | 3 | 4 | 27 | B |

*Knowledge Educator Systematic Review*

| **Author:** | **Abstract & title:** | **Introduction & aims:** | **Method & data:** | **Sampling:** | **Data analysis:** | **Ethics & bias:** | **Results:** | **Transferability or generalizability:** | **Implications & usefulness:** | **Total:** | **Grade:** |
| --- | --- | --- | --- | --- | --- | --- | --- | --- | --- | --- | --- |
| AB InBev, 2018 | 4 | 4 | 3 | 1 | 2 | 1 | 3 | 4 | 4 | 26 | B |
| Abdool-Gaffar, *et al., 2011* | 4 | 4 | 4 | 1 | 1 | 1 | 2 | 4 | 4 | 25 | B |
| Ahmed, *et al., 2015* | 4 | 4 | 4 | 3 | 4 | 4 | 4 | 4 | 4 | 35 | A |
| Astra Zeneca, n.d. | 2 | 4 | 1 | 1 | 1 | 1 | 3 | 3 | 4 | 20 | C |
| AVON Global Scholars, n.d. | 1 | 4 | 1 | 1 | 1 | 1 | 3 | 3 | 4 | 19 | C |
| Laar, et al., 2019 | 3 | 3 | 4 | 3 | 4 | 3 | 4 | 3 | 4 | 31 | A |
| Malekzadeh, et al. 2020 | 4 | 4 | 2 | 2 | 2 | 1 | 2 | 3 | 4 | 24 | C |
| Merck KGaA, 2021 | 4 | 4 | 2 | 2 | 1 | 1 | 1 | 4 | 4 | 23 | C |
| Novo Nordisk, 2017 | 4 | 4 | 2 | 1 | 2 | 1 | 2 | 4 | 4 | 24 | C |
| Pastakia, *et al., 2020* | 4 | 4 | 4 | 3 | 4 | 3 | 4 | 4 | 4 | 34 | A |
| Patel, *et al., 2010* | 4 | 4 | 4 | 4 | 3 | 3 | 4 | 3 | 4 | 33 | A |
| Pfizer Upjohn, 2020 | 1 | 4 | 2 | 1 | 2 | 1 | 3 | 3 | 4 | 22 | C |
| Ratzan, 2013 | 4 | 4 | 4 | 3 | 4 | 2 | 4 | 2 | 4 | 31 | B |
| Reeves, 2011 | 4 | 4 | 2 | 1 | 1 | 1 | 1 | 3 | 4 | 21 | C |

*Investment and Finance Systematic Review*

| **Author:** | **Abstract & title:** | **Introduction & aims:** | **Method & data:** | **Sampling:** | **Data analysis:** | **Ethics & bias:** | **Results:** | **Transferability or generalizability:** | **Implications & usefulness:** | **Total:** | **Grade:** |
| --- | --- | --- | --- | --- | --- | --- | --- | --- | --- | --- | --- |
| Allen, 2017 | 4 | 3 | 3 | 3 | 1 | 3 | 4 | 3 | 4 | 28 | **B** |
| Anson et al., 2012 | 4 | 4 | 4 | 4 | 4 | 1 | 4 | 4 | 4 | 33 | **A** |
| Armstrong-Hough et al., 2020 | 4 | 3 | 4 | 3 | 4 | 1 | 4 | 4 | 4 | 31 | **A** |
| Ashigbie et al., 2020 | 4 | 4 | 4 | 4 | 4 | 4 | 4 | 4 | 4 | 36 | **A** |
| Beran et al., 2019 | 4 | 3 | 1 | 1 | 1 | 1 | 4 | 3 | 3 | 21 | **C** |
| Bloom et al., 2011 | 4 | 4 | 3 | 3 | 3 | 1 | 3 | 3 | 3 | 27 | **B** |
| Cameron et al., 2009 | 4 | 4 | 4 | 4 | 4 | 1 | 3 | 4 | 4 | 32 | **A** |
| Cattaneo & Piemonte, 2021 | 3 | 2 | 2 | 1 | 1 | 1 | 3 | 2 | 3 | 18 | **C** |
| Dugee et al., 2018 | 4 | 4 | 4 | 3 | 4 | 2 | 3 | 3 | 4 | 31 | **A** |
| Dutta & Ly, 2018 | 1 | 1 | 1 | 1 | 1 | 1 | 3 | 1 | 3 | 13 | **C** |
| El-Saharty S, et al., 2013 | 1 | 4 | 1 | 3 | 1 | 1 | 4 | 4 | 4 | 23 | **C** |
| Ganju et al., 2020 | 3 | 4 | 3 | 1 | 1 | 1 | 4 | 2 | 3 | 22 | **C** |
| Heidari et al., 2019 | 4 | 4 | 4 | 3 | 4 | 1 | 4 | 4 | 4 | 32 | **A** |
| Holt et al., 2017 | 2 | 2 | 1 | 3 | 1 | 1 | 3 | 3 | 3 | 19 | **C** |
| Jones, 2021 | 4 | 4 | 3 | 3 | 2 | 1 | 4 | 4 | 4 | 29 | **B** |
| Kanzler & Ng, 2012 | 1 | 4 | 1 | 3 | 1 | 1 | 4 | 4 | 4 | 23 | **C** |
| Khuluza & Haefele-Abah, 2019 | 4 | 3 | 4 | 4 | 4 | 4 | 4 | 4 | 4 | 35 | **A** |
| Kishore et al., 2015 | 3 | 3 | 1 | 1 | 1 | 1 | 3 | 2 | 3 | 18 | **C** |
| Ladusingh L et al., 2018 | 4 | 4 | 4 | 3 | 4 | 1 | 3 | 3 | 3 | 29 | **B** |
| Lambert & Kolbe-Alexander, 2013 | 3 | 3 | 2 | 3 | 1 | 3 | 4 | 3 | 4 | 26 | **B** |
| Lekshmi et al., 2014 | 4 | 4 | 2 | 4 | 3 | 1 | 3 | 3 | 4 | 28 | **B** |
| Mendis & Chestnov, 2013 | 4 | 3 | 3 | 3 | 2 | 1 | 4 | 3 | 4 | 27 | **B** |
| Mendis S, et al., 2007 | 4 | 3 | 4 | 4 | 4 | 1 | 4 | 4 | 4 | 32 | **A** |
| Mhlanga & Suleman, 2014 | 4 | 4 | 4 | 4 | 4 | 4 | 4 | 4 | 4 | 36 | **A** |
| Moodie et al., 2013 | 4 | 3 | 2 | 1 | 1 | 1 | 3 | 2 | 3 | 20 | **C** |
| Prescott & Stibbe, 2017 | 1 | 4 | 3 | 1 | 1 | 1 | 4 | 2 | 4 | 21 | **C** |
| Rahman et al., 2013 | 4 | 4 | 4 | 4 | 4 | 4 | 4 | 4 | 4 | 36 | **A** |
| Sado & Sufa, 2016 | 4 | 4 | 4 | 4 | 4 | 4 | 3 | 4 | 3 | 34 | **A** |
| Schmutz et al., 2019 | 3 | 3 | 4 | 4 | 4 | 1 | 3 | 3 | 3 | 28 | **B** |
| Singh et al., 2016 | 4 | 2 | 4 | 4 | 4 | 4 | 3 | 3 | 3 | 31 | **A** |
| Subramaniam et al., 2018 | 4 | 4 | 3 | 3 | 4 | 3 | 3 | 3 | 4 | 31 | **A** |
| Tripathy & Prasad, 2018 | 4 | 2 | 4 | 4 | 4 | 4 | 4 | 4 | 4 | 34 | **A** |
| Tusubira et al., 2020 | 4 | 2 | 4 | 4 | 4 | 4 | 4 | 2 | 4 | 32 | **A** |
| Shellaby & Henshall, 2018 | 4 | 4 | 1 | 2 | 1 | 1 | 4 | 2 | 4 | 23 | **C** |
| UNICEF, 2019 | 2 | 4 | 1 | 1 | 1 | 1 | 4 | 2 | 4 | 20 | **C** |
| FAO et al., 2020 | 4 | 3 | 3 | 3 | 2 | 1 | 4 | 4 | 4 | 28 | **B** |
| van Mourik et al, 2010 | 4 | 4 | 3 | 4 | 4 | 1 | 4 | 4 | 4 | 32 | **A** |
| World Bank, 2013 | 4 | 4 | 2 | 3 | 1 | 1 | 4 | 4 | 4 | 27 | **B** |
| WHO, 2013 | 1 | 3 | 2 | 3 | 1 | 1 | 4 | 4 | 4 | 23 | **C** |
| WHO, 2019 | 1 | 4 | 2 | 1 | 1 | 1 | 4 | 2 | 4 | 20 | **C** |
| The Advisory Group on the Governance of the Private Sector for UHC, 2020 | 3 | 4 | 3 | 3 | 1 | 1 | 4 | 4 | 4 | 27 | **B** |
| You et al., 2019 | 4 | 4 | 3 | 2 | 3 | 1 | 4 | 2 | 3 | 26 | **B** |
